# Cancer incidence and mortality among patients with new-onset atrial fibrillation: A population-based matched cohort study

**DOI:** 10.1016/j.neo.2024.101080

**Published:** 2024-11-08

**Authors:** Nadine Zakkak, Matthew Barclay, Arturo Gonzalez-Izquierdo, Amand Floriaan Schmidt, Gregory Y.H. Lip, Georgios Lyratzopoulos, Rui Providencia

**Affiliations:** a Epidemiology of Cancer Healthcare and Outcomes Group (ECHO), Department of Behavioural Science and Health, Institute of Epidemiology and Health Care, University College London, London, United Kingdom; b Cancer intelligence, Cancer Research UK, London, United Kingdom; c Centre for Health Data Science, Institute of Applied Health Research, University of Birmingham, United Kingdom; d Institute of Health Informatics, University College London, United Kingdom; e Institute of Cardiovascular Science, Faculty of Population Health, University College London, London, United Kingdom; f UCL British Heart Foundation Research Accelerator, London, United Kingdom; g Department of Cardiology, Amsterdam Cardiovascular Sciences, Amsterdam University Medical Centres, University of Amsterdam, Amsterdam UMC, the Netherlands; h Department of Cardiology, Division Heart and Lungs, University Medical Center Utrecht, Utrecht University, Utrecht, the Netherlands; i Liverpool Centre for Cardiovascular Science at University of Liverpool, Liverpool John Moores University and Liverpool Heart & Chest Hospital, Liverpool, United Kingdom; j Danish Center for Health Services Research, Department of Clinical Medicine, Aalborg University, Aalborg, Denmark; k Barts Heart Centre, St Bartholomew's Hospital, London, United Kingdom

**Keywords:** Atrial fibrillation, Cancer, Diagnosis, Incidence, Risk, Mendelian randomisation

## Abstract

**Background:**

Understanding the risk of cancer after the diagnosis of another condition can present opportunities for earlier diagnosis. We examined the risk of cancer diagnosis conditional on prior diagnosis of atrial fibrillation (AF).

**Methods:**

Linked electronic health records were used to identify patients aged ≥18 with new-onset AF and age-sex-matched controls. Cumulative incidence of and mortality from cancer (overall and cancer-site specific) within three months, three months to five years and beyond five years from diagnosis of AF were examined. Findings were further validated using Mendelian randomisation (MR).

**Results:**

The cohort included 117,173 patients with new-onset AF and 117,173 matched controls (median age 78). In the first three months, 2.2% of AF patients were diagnosed with cancer vs. 0.47% in controls (relative risk: 4.7 [95%CI 4.2-5.4] in men and 4.4 [95%CI 3.8-5.0] in women). Nearly 80% of cancers related to thoracic or abdominal organs. Differences in cumulative incidence were only evident in women between three months and five years (subdistribution hazard ratio=1.1 [95%CI 1.01-1.12]) and absent in all patients beyond five years. MR analysis did not support the presence of a causal association between AF and major cancer subtypes.

**Conclusion:**

There is a large short-term increase in cancer incidence and mortality following new-onset AF. The findings may reflect incidental identification of AF or paraneoplastic manifestation. New-onset AF confers high short-term risk of cancer diagnosis, at levels comparable with symptomatic risk threshold mandating urgent assessment for suspected cancer.

## Highlights

• Temporal associations between new-onset AF and cancer were examined.
• New-onset AF is associated with an increased short-term cancer risk.
• New-onset AF with possible cancer symptoms should raise cancer suspicion.
• No evidence for an association between AF and cancer risk in the longer term.
• Mendelian randomisation indicated no shared (AF-cancer) genetic predisposition.

## Introduction

Improvements in chronic disease management mean more patients are developing multiple conditions. Atrial fibrillation (AF) is a common cardiac arrhythmia predicted to affect 17.9 million individuals in the European Union and UK by 2060.[1] Associations between AF and other cardiovascular conditions are well-established, but associations with other conditions, including cancer, have also been reported.[[2], [3], [4], [5]] Understanding inter-related risks between diseases is important, though responsible mechanisms and clinical implications vary by length of follow-up. Shortly after being diagnosed with AF, a patient may be diagnosed with cancer, which might have remained asymptomatic or formed part of the syndromic presentation that led to the AF diagnosis.[6] In the longer term, a patient with AF may be at risk of developing cancer due to shared risk factors. Understanding short-term associations is important to support improvements in the diagnostic management of chronic conditions, while understanding long-term associations is important for public health and preventive actions.

Prior work has considered the risk of a subsequent diagnosis of cancer after new-onset AF.[[6], [7], [8], [9]] Although previous studies reported an increased risk of cancer within the first three months after new-onset AF, an all-women study and a study in the Danish population reported an increased risk of cancer up to a year later.[6,9] While some studies have reported increased risk of lung,[8,9] colon,[6,8,9] and breast[8] cancers after new-onset AF, others have found no such association.[6,7,9] The reported associations between AF and cancer remain highly inconsistent. Previous studies had a small sample size, were restricted to a single sex of patients, lacked a control group, or did not adjust for potential confounders. Definitive assessment of the association between new-onset AF and cancer is required.

This study aims to elucidate whether there is temporal association between new-onset AF and cancer by characterising the risk of mortality from cancer and incidence of cancer overall, and of specific types of cancer across a follow-up time split into three time periods.

## Methods

### Risk of cancer

#### Data and study population

Data on 6,529,382 patients in England were provided by the Clinical Practice Research Datalink (CPRD) Gold between 1^st^ January 1998 and 31^st^ May 2016 linked to the national cancer registry, hospital data (Hospital Episode Statistics, HES) and death registry data from the Office for National Statistics (ONS).

#### Study design

We conducted a population-based retrospective cohort study of patients with new-onset AF and age-sex-matched controls without AF at the start of their follow-up.

AF was identified in CPRD using Read Codes G573200, G573400, G573500, 3272.00, G573000, G573300, G573.00, G573z00 and HES using International Classification of Disease, tenth edition (ICD-10) “I48” and its subdomains, following the CALIBER phenotype.[10] We used the earliest record of AF diagnosis in either CPRD or HES.

#### Analysis cohort

The cases comprised patients aged 18 to 100 years presenting with new-onset AF in primary or secondary care between 1^st^ January 1998 and 31^st^ December 2014. New-onset AF was only considered if the patient had not had cancer or AF history records in previous years and if the diagnosis occurred after the patient had been registered at the practice for at least one year, the practice was viewed as up-to-standard by CPRD and before the patient's transfer out date, death, and the practice's last data collection date. The earliest eligible new-onset AF date was chosen as the start date for each patient.

Cases were followed-up until the earliest of their 31^st^ December 2015, cancer incidence or mortality outcomes (see Outcomes below) or intervening non-cancer death.

The controls comprised patients aged 18 to 101 years with their start of follow-up defined as the latest of 1^st^ January 1998, the date when the patient had been registered at the practice for at least one year and the practice was viewed up-to-standard by CPRD. Patients were only considered if they had not had cancer or AF history records before their start date. Patients were eligible to be controls prior to becoming cases.

Controls were followed-up until the earliest of 31^st^ December 2015, diagnosis of new-onset AF, cancer incidence or mortality outcomes (see Outcomes below) or intervening non-cancer death.

Cases were 1-1 matched with controls based on sex, year of birth (allowing for a 1-year difference), and follow-up period (AF diagnosis occurs within control's follow-up period). The date of AF diagnosis of the matched case was used as the index date for their corresponding control.

#### Covariates

We considered age, sex, smoking status, alcohol consumption status, diabetes, and hypertension to be potential risk factors for both AF and cancer and adjusted for all or some of the covariates in our analyses.[[11], [12], [13], [14]] We did not adjust for obesity because of the high rate of missingness in our data (>50%). CALIBER phenotypes were used to identify the covariates recorded at baseline (on or before start of follow-up) based on the CPRD and HES records.[10] Details on how the covariates were identified are available in Supplemental texts S1-S4 and Supplemental tables S1-S4.

#### Outcomes

Our two primary outcomes were (1) cancer incidence identified from the national cancer registry and (2) cancer mortality identified from the ONS. Secondary outcomes included specific cancer incidence by eight body regions (abdomen, brain/central nervous system, chest, head and neck, pelvis, upper and lower limbs, unknown primary, and other cancers) and 12 organ systems (breast, cardiovascular, digestive, endocrine, immune and haematological, nervous, reproductive, respiratory, skeletal, urinary, unknown primary, and other cancers). Each category of body region/organ system was considered independently of other categories (e.g. if a patient had cancer in the respiratory system first then cancer in the urological system at a later date, both outcomes would be considered in their corresponding categories). A full list of ICD-10 codes can be found in Supplemental table S5.

#### Analysis

We examined associations between new-onset AF and cancer in the short-term (up to three months follow-up), medium-term (three months to five years) and long-term (beyond five years). These splits were necessary due to evidence of violation of the proportional sub-distribution hazards assumption and allowed us to assess changes in associations over follow-up. All outcomes were examined in each follow-up period.

For short-term follow-up analysis, sex-stratified Poisson regression was used, with robust standard errors. This was reasonable as few patients were censored within three months. For medium and long follow-up analysis, sex-stratified Fine-Gray regression analysis[15] was used to account for censoring and competing risks (i.e. all-cause death and other cause death for the cancer incidence and cancer mortality outcomes, respectively). All models were adjusted for age, diabetes, hypertension, and smoking status. Models with cancer of the digestive system as outcome were additionally adjusted for alcohol consumption status. Outcomes that occurred in ≤0.04% of patients for each of cases and controls in the first three months were excluded from any further analysis: brain/CNS, upper and lower limbs, cardiovascular, endocrine, nervous, and skeletal cancers in both men and women and breast cancer in men.

Data management and statistical analysis, except for Fine-Gray regression analysis, were conducted in R version 4.1.2. Analysis made use of the following R packages: tidyverse version 2.0.0[16] and tidycmprsk version 0.2.0.[17] Fine-Gray regression analysis was conducted in Stata version 16 using the stcrreg command. This study followed the Strengthening the Reporting of Observational Studies in Epidemiology (STROBE) guideline for reporting (Appendix).

### Mendelian randomisation analyses

Mendelian randomisation (MR) was employed to estimate the potential causal effect of the liability of AF had on the occurrence of cancers, specifically lung cancer (29,266 cases),[18] breast cancer (133,384 cases),[19] and colorectal cancer (73,149 cases).[20] Given that genetic variants are determined during gametogenesis, MR is protected against confounding and reverse causation, and may therefore provide a robust indication of potential causality.

Genetic variants on AF (exposure) were sourced from Nielsen et al., (2018) (60,620 cases[21]), utilising an R-squared of 0.05 linkage disequilibrium (LD) threshold, indicating pairwise correlation between genetic variants, a minor allele frequency of 0.01 or larger and an F-statistic of 24. LD was calculated based on a random sample of 5,000 UK Biobank reference participants.

MR estimates were derived using the inverse-variance weighted (IVW) and Egger methods accounting for residual correlation using the aforementioned UK Biobank reference data. An h model selection framework by Bowden et al., (2017) was utilised to determine which estimator (IVW or Egger) was supported by the available genetics data.[22] Variants with high leverage and/or outlier statistics were removed to limit horizontal pleiotropy bias.

## Results

### Risk of cancer

#### Cohort description

**Fig. 1:**
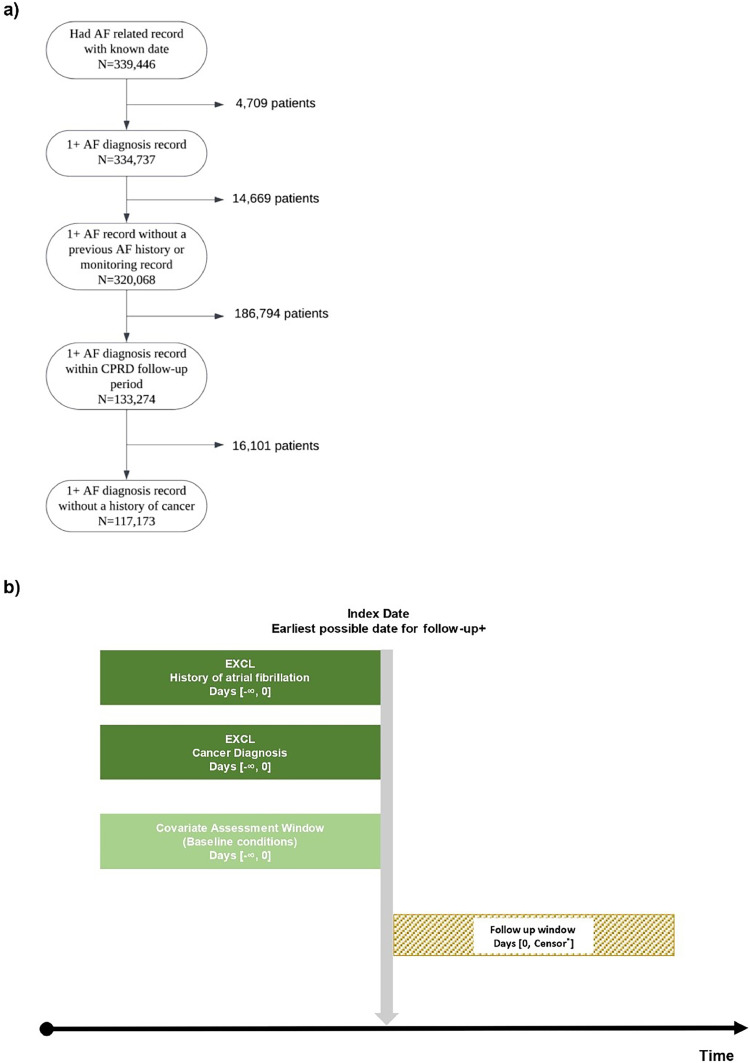
Identification of cohort. This diagram shows the process of identification of a) cases b) controls. Patients that shared the same tumour ID identified in the cancer registry were excluded from analysis. ^+^Start of follow-up latest of 1st January 1998, patient turned 18 years old, the date when the patient had been registered at the practice for at least one year and the practice was viewed up-to-standard by CPRD. *Censored at minimum of 31st December 2015, new-onset atrial fibrillation diagnosis, cancer or death.

The analysis cohort comprised 117,173 patients with new-onset AF and 117,173 matched controls (Fig 1). Half (50.6%) of patients were men with a median age of 75 years (IQR 66-82) at index; women were older with a median age of 81 years (IQR 74-87) (Table 1). Patients without AF had longer follow-up periods compared to patients with AF (Table 2). In the cancer mortality analysis, across all outcomes (i.e., cancer death, other cause death, censoring), men with AF were followed-up for a median of 4.1 years vs 5.4 years without AF, while women had shorter follow-up periods (3.4 years with AF vs 5.0 years without AF).

**Table 1:** Baseline characteristics of patients with new-onset atrial fibrillation (AF) and their matched controls, stratified by men and women.

		Men		Women	
		Control	AF	Control	AF
Total		59258	59258	57915	57915
Age in years	Median (IQR)	74.5 (65.6, 81.7)	74.6 (65.7, 81.8)	80.8 (73.4, 86.8)	80.8 (73.5, 86.8)
Index of Multiple Deprivation (2015)	1 - Least deprived	9899 (16.7%)	9923 (16.7%)	9582 (16.5%)	9337 (16.1%)
	2	11050 (18.6%)	11009 (18.6%)	10942 (18.9%)	10670 (18.4%)
	3	11587 (19.6%)	11593 (19.6%)	11525 (19.9%)	11464 (19.8%)
	4	13501 (22.8%)	12861 (21.7%)	13344 (23.0%)	12649 (21.8%)
	5 - Most deprived	13221 (22.3%)	13872 (23.4%)	12522 (21.6%)	13795 (23.8%)
Diabetes		6882 (11.6%)	10405 (17.6%)	5534 (9.56%)	8185 (14.1%)
Hypertension		21906 (37.0%)	33651 (56.8%)	27269 (47.1%)	37703 (65.1%)
Alcohol status	Current	38590 (65.1%)	42808 (72.2%)	30689 (53.0%)	31443 (54.3%)
	Ex	1120 (1.89%)	1755 (2.96%)	921 (1.59%)	1389 (2.40%)
	Missing	12876 (21.7%)	7666 (12.9%)	12467 (21.5%)	9598 (16.6%)
	Non	6672 (11.3%)	7029 (11.9%)	13838 (23.9%)	15485 (26.7%)
Ex/current smoker		35034 (59.1%)	41978 (70.8%)	26510 (45.8%)	30983 (53.5%)

**Table 2:** Follow-up time and proportion of main outcomes in cancer death analysis and cancer incidence analysis for patients with new-onset atrial fibrillation (AF) and their matched controls, stratified by men and women.

			Men				Women		
Analysis			Control		AF		Control		AF
*Cancer death*									
	Median follow-up time (IQR)^a^		5.43 (2.79-9.04)		4.12 (1.58-7.79)		4.95 (2.49-8.43)		3.40 (1.05-6.82)
	Proportion of patients that died of cancer after index								
	≤ 3 months^b^		52 (0.09%)		509 (0.86%)		59 (0.10%)		447 (0.77%)
	3 months - 5 years^c^		1950 (3.34%)		2541 (4.81%)		1654 (2.92%)		1869 (3.81%)
	> 5 years^d^		1584 (4.99%)		1505 (5.93%)		1138 (3.97%)		940 (4.43%)
*Cancer incidence*									
	Median follow-up time (IQR)^a^		5.11 (2.59-8.71)		3.85 (1.38-7.43)		4.77 (2.36-8.21)		3.22 (0.92-6.57)
	Proportion of patients that were diagnosed with cancer after index								
	≤ 3 months^b^		293 (0.49%)		1422 (2.40%)		252 (0.44%)		1106 (1.91%)
	3 months - 5 years^c^		4287 (7.37%)		4194 (8.07%)		3061 (5.41%)		2990 (6.16%)
	> 5 years^d^		2573 (8.53%)		2282 (9.52%)		1724 (6.23%)		1346 (6.63%)

a **Median follow-up time (Interquartile range) to any of the possible outcomes**

b Denomintor: all patients

c Denominator: patients with no outcomes up to 3 months following index date.

d Denominator: patients with no outcomes up to 5 years following index date.

**Fig. 2:**
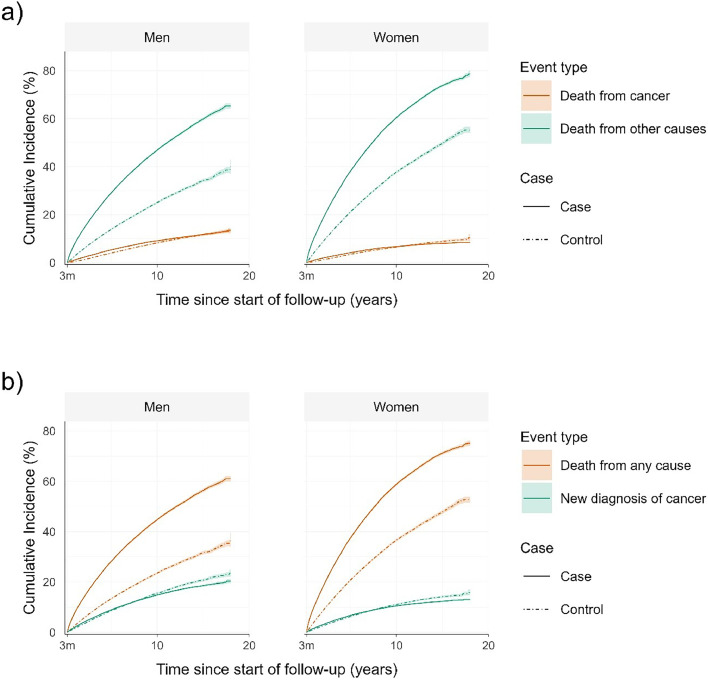
Cumulative incidence curves of (a) death from cancer and other causes (b) cancer incidence and death from any cause.

**Fig. 3:**
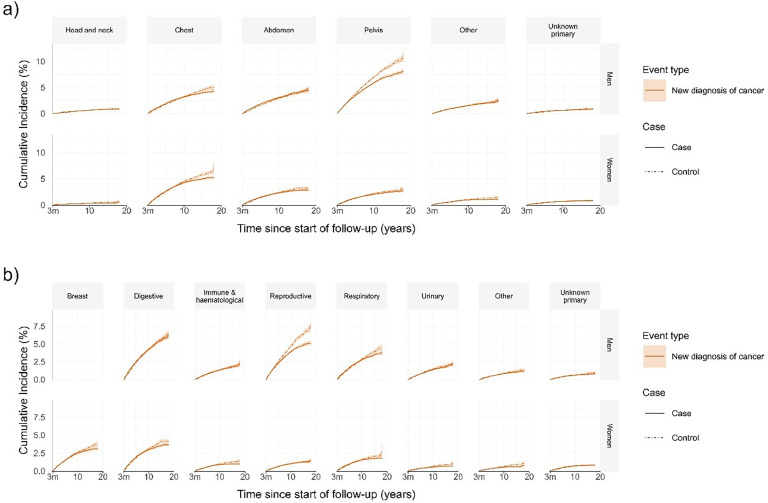
Cumulative incidence curves of cancer incidence by (a) anatomical region (b) organ system. (a) Cumulative incidence curve of new diagnosis of cancer by anatomical region in patients with new-onset atrial fibrillation (AF) and their matched controls starting at 3 months after index date. Only cancer incidence curve is shown for simplification (i.e. its competing risk death from any cause – has been omitted from the visual). New diagnosis of cancer in studied anatomical regions was similar, or slightly lower, in patients with AF compared to controls beyond 3 months of follow-up. (b) Cumulative incidence curve of new diagnosis of cancer by organ system in patients with new-onset atrial fibrillation (AF) and their matched controls starting at 3 months after index date. Only cancer incidence curve is shown for simplification (i.e. its competing risk death from any cause – has been omitted from the visual). New diagnosis of cancer in studied organ systems was similar, or slightly lower, in patients with AF compared to controls beyond 3 months of follow-up. Shaded regions represent 95% confidence intervals. 3m = 3 months.

Following new-onset of AF, 13,340 patients were diagnosed with cancer: 2528 in the first three months, 7184 after three months and less than five years, and 3628 after the initial five years (Table 2). When accounting for censoring, cumulative incidence of cancer was 7.9% at five years and 12.7% at ten years (Supplemental Fig. S1). Cancer deaths and cancer incidence appeared higher in patients with AF compared to patients without AF in both men and women up to five years following the index date (Fig 2). The incidence of cancer by all studied anatomical regions and organ systems was similar in patients with and without AF beyond three months of follow-up, or slightly lower, such as cancer in the chest after ten years of follow-up (Fig 3).

(a) Cumulative incidence curves of death from cancer and death from other causes in patients with new-onset atrial fibrillation (AF) and their matched controls starting at 3 months after index date. Death from cancer was higher in patients with AF compared to controls up to 5 years after index date, while death from other causes remained higher in patients with AF compared to controls up to 18 years after index date. (b) Cumulative incidence curves of new diagnosis of cancer and death from any cause in patients with new-onset atrial fibrillation (AF) and their matched controls starting at 3 months after index date. New diagnosis of cancer was higher in patients with AF compared to controls up to 5 years after index date, while death from any cause remained higher in patients with AF compared to controls up to 18 years after index date. Shaded regions represent 95% confidence intervals. 3m = 3 months.

### Short-term

**Fig. 4:**
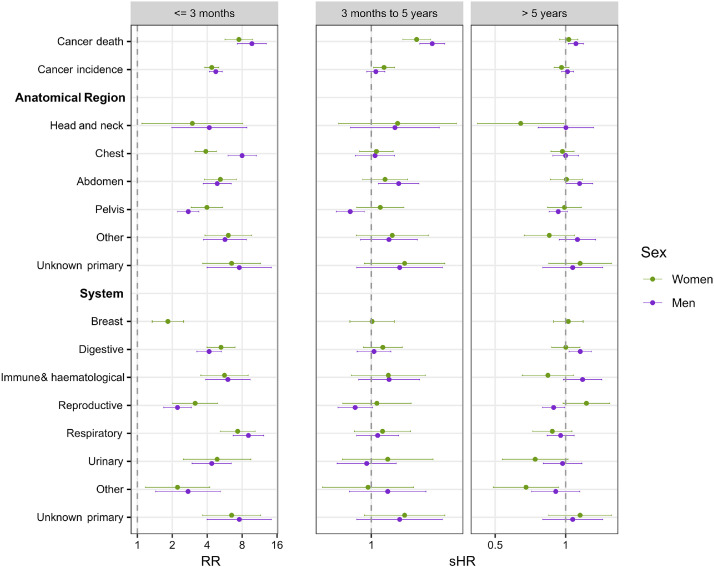
Adjusted model estimates of all outcomes. Adjusted model estimates of all outcomes: cancer death, any cancer incidence, cancer incidence by anatomical regions and by organ systems. All models were adjusted for age, diabetes, hypertension, and smoking status. Digestive system cancer model was additionally adjusted for alcohol drinking status. ≤3 months (short-term) estimates represent risk ratios (RR) from adjusted Poisson regression. 3 months to 5 years (medium-term) and >5 years (long-term) estimates represent sub-distribution hazard ratios (sHR) from adjusted Fine-Gray regression. Risk of cancer in AF patients is elevated in the short-term compared to their matched controls. Risk of cancer death remains elevated in AF patients compared to controls up to 5 years post-diagnosis.

In the first three months, all studied outcomes, cancer death and cancer incidence (overall and specific sites), occurred more frequently in patients with incident AF compared to patients without AF (Table 2; Supplemental table S6). 2.4% [95% CI 2.28-2.53] of men with AF were diagnosed with cancer within 3 months of their AF diagnosis, compared to 0.5% [95% CI 0.44-0.55] of matched controls. Adjusted risk ratios for cancer death and cancer incidence (overall and cancer-site specific) were higher in patients with new-onset AF compared to their controls in the first 3 months following diagnosis/index (Fig 4; Supplemental table S7). For example, for men, the risk of cancer death was ten times higher in cases than controls (risk ratio (RR) 9.7, [95% CI 7.3-13.0]), while risk of cancer incidence was five times higher (RR 4.7, [95% CI 4.2-5.4]); results for women were broadly similar. There was excess risk in cases for all cancers of any region or system, although risks were particularly elevated for respiratory cancers and cancers of unknown primary site in both sexes, while the association was weaker for female breast cancer and for reproductive / pelvic cancers in both sexes.

Analysis of cancer location by body region showed that 79.2% of diagnosed cancers in patients with new-onset AF in this time-period were located in the chest, abdomen, or pelvis (Supplemental Fig. S2). System-wise, the majority of cancers in patients with new-onset AF involved the respiratory (28.4%), digestive (27.6%), blood and immune (10.2%) and reproductive system (10.1%) (Supplemental Fig. S3).

#### Medium-term

Cumulative incidence of cancer death was higher in both men and women with incident AF compared to patients without AF from three months up to five years following diagnosis/index (subdistribution hazard ratio (sHR) = 1.4 [95% CI 1.3-1.4] in men vs 1.3 [95% CI 1.2-1.3] in women) **(**Fig. 4; Supplemental table S7). Cumulative incidence of cancer diagnosis in women was slightly increased in patients with AF compared to patients without AF from three months up to five years following diagnosis/index (sHR = 1.1 [95% CI 1.0-1.1]), and there was no apparent difference by presence of AF in men. There was no apparent difference in cumulative incidence for cancers of any region or system in patients with and without AF, except for an increase in cumulative of cancer in the abdominal region in men with AF compared to men without AF (sHR = 1.1 [95% CI 1.04-1.26]) and a slight decrease in cumulative incidence of cancer in the pelvis region in men with AF compared to men without AF (sHR = 0.90 [95% CI 0.84 – 0.97]).

#### Long-term

Beyond five years, for most comparisons there was no apparent difference in cumulative incidence of cancer death or cancer incidence (overall and cancer-site specific). There were six exceptions, although differences were small (Fig. 4; Supplemental table S7). The cumulative incidence of cancer death in men with AF was slightly higher than men without AF (sHR = 1.1 [95% CI 1.0-1.2]). There was limited evidence of an increase in risk of cancer in the abdominal region and digestive system in men (sHR = 1.2 [95% CI 1.0-1.3] in both) and a decrease in risk of cancer in the reproductive system in men, cancer in the head and neck in women and cancer in other organ systems in women (sHR = 0.89 [95% CI 0.80-0.99]; sHR = 0.64 [95% CI 0.42-0.98]; sHR = 0.68 [95% CI 0.49-0.93], respectively).

### Mendelian randomisation analysis

Mendelian Randomisation analyses did not confirm the presence of a causal association between AF and three investigated cancers: lung cancer (OR = 1.00 [95%CI 0.96-1.03]; 29,266 cases & 56,450 controls), breast cancer (OR = 0.95 [95%CI 0.81-1.11]; 133,384 cases & 113,789 controls), and colorectal cancer (OR = 1.02 [95%CI 0.99-1.04]; 73,149 cases & 112,849 controls) (Supplemental table S8).

## Discussion

### Summary

In this study, we report the cumulative incidence of cancer, overall and of specific types, and of cancer death in three time-periods following AF diagnosis: initial three months, three months up to five years and beyond five years. The vast majority of cancers diagnosed following an AF presentation were located in the chest, abdomen, or pelvis.

Our principal findings are as follows: (i) Risk of cancer was high immediately following a new diagnosis of AF but rapidly became similar to the risk of cancer in controls. Compared to their controls, patients with incident AF had a higher risk of cancer following AF diagnosis within the first three months in both men and women, and between three months and five years in women, without a difference in the risk of cancer in patients with and without AF between three months and five years in men and beyond five years in both men and women. Our Mendelian Randomisation analyses does not support the presence of a causal association between AF and lung, breast or colorectal cancer; and (ii) The cumulative incidence of cancer death remained increased in men with AF beyond five years following diagnosis compared to patients without AF, while the cumulative incidence was increased in women up to five years following AF and there was no difference in risk beyond five years.

#### Comparison with literature

Using population-based data in England, we studied the association between AF and cancer, with a wide range of cancer type outcomes examined. We have comprehensively looked at risk of different cancer types as per organ location, organ system and location in main anatomic areas. Furthermore, we assessed causality between AF and three common cancer sites.

Similar to previous studies, we found an increased risk of cancer following an incident AF diagnosis that weakened over time.[6,8,9] The highest risk of cancer appeared to be within the first three months following AF diagnosis, with two studies reporting an increased risk up to one year after AF diagnosis,[6,9] while we have found a slight increase in risk up to five years following AF in women and no difference in risk in men beyond three months. In concordance with a previous study, the Mendelian Randomisation analyses did not confirm a causal association between AF and lung, breast or colorectal cancer.[23]

We also observed that AF was associated with a ten-fold and eight-fold increased risk of cancer death in men and women, respectively up to three months post AF diagnosis compared to patients without AF. The risk of cancer death in patients with incident AF diminished over time, and there was no difference in cumulative incidence of cancer death in women beyond five years. Similarly, Conen et al. (2016) reported a slightly increased risk of cancer death up to a year post AF diagnosis, but not over the entire follow-up period.[6]

We found a steady increase in risk for all cancer subtypes within the three months following diagnosis which was no longer significant beyond three months, except for cancer in the abdominal region in men between three months and five years and beyond five years, and cancer in the digestive system beyond five years. A study from the Danish population found an increased risk of cancer across varied subtypes within the first three months and beyond three months following AF diagnosis, although without adjustment for potential confounders.[8] An all-women study found an increased risk of colon cancer after incident AF, but not for breast or lung cancer, across the entire follow-up period.[6] A different Danish analysis reported increased risk of colorectal and lung cancers in both men and women, up to a year after AF diagnosis, with the highest risk within the first three months but without associations with breast and prostate cancers throughout the follow-up period.[9] On the other hand, a four-year study in the United States did not find an association between AF and colorectal cancer but an increased risk of breast cancer, possibly attributed to digoxin treatment.[7]

#### Interpretation and clinical relevance

Our study is not equipped to provide complete aetiological proof of the observed association. However, we can consider three principal potential mechanisms, comprising two non-causal and one causal explanation. First, the increased risk of cancer, overall and all studied types, within the first three months after AF diagnosis that decreases and disappears over time suggests the possibility of incidental identification of asymptomatic cancer after presentation with AF symptoms. Such incidental identification is unlikely to be dominant given the strong association with short-term cancer mortality; incidentally diagnosed cancers are typically early stage and have good short-term prognosis. Second, given diagnosis of AF only requires an electrocardiogram, but histological diagnosis of cancer is contingent upon further referrals, specialist investigations, and pathological examination of biopsy specimens, there is a possibility of reverse causality through identification of asymptomatic / well-tolerated AF after presentation with underlying cancer symptoms with recorded diagnosis happening in the opposite order. Finally, findings from our Mendelian randomisation analyses do not support the causality of the association of AF with subsequently diagnosed cancer. On the other hand, it is possible that AF may be a paraneoplastic syndrome manifestation of underlying cancer.[24] The literature suggests various pathophysiological mechanisms of cancer-induced AF such as cancer-related inflammation, cancer-related autonomous dysregulation, metabolic and electrolyte abnormalities, and infections.[25]

Patients with incident AF and clinical presentation suggestive of cancer should be investigated for underlying cancer, especially in the first months following AF diagnosis. Due to the observed locations and involved organ systems, a CT chest-abdomen-pelvis accompanied by a complete blood count with peripheral blood smear, upper gastrointestinal endoscopy and colonoscopy (for patients with anaemia) and a breast ultrasound for women, would likely diagnose >80% of cancers. Further research is needed to characterise the population of new-onset AF patients to examine whether there are opportunities for earlier diagnosis of cancer, and to establish the most effective set of investigations.

#### Strengths and limitations

The study's strengths are the use of large England population-based sample, examination of both short- and longer-term follow-up periods, the use of Fine-Gray regression analysis taking into consideration the competing risk of death and censoring over long follow-up periods and the wide range of cancer type outcomes examined. Data from CPRD have high completeness of clinical information recorded [26,27], and are generally representative of the age, gender and geographic distribution of the UK population.[28] While the data relate to UK patients, there are no comparable population-based data sources incorporating information from primary care and hospital data in other countries, nonetheless the disease associations we describe are likely to be overall generalisable to a great degree.

Using electronic health records, we were able to obtain a large sample size of AF cases and their matched controls. However, some AF cases might have been misclassified due to coding, despite our use of a validated AF phenotype.[29] Accounting for more detailed alcohol and smoking statuses is likely to improve the models because the quantity and duration of alcoholic drinking and smoking might change the risks of AF and cancer; however, these are difficult to capture in electronic health records.[12] As for any observational study, there is always a risk of unmeasured confounders (e.g. potentially carcinogenic agents that did not occur at random across AF patients and controls).

## Conclusion

In conclusion, there is a large increase in risk of cancer incidence and mortality in the first three months following AF diagnosis, particularly for cancers of respiratory and abdominal organs and of unknown primary site. However, no associations are observed in the medium- to long-term, and there is no evidence of shared genetic predisposition. Short-term associations can reflect incidental identification of AF, or paraneoplastic manifestations. New-onset AF should raise suspicion of underlying cancer, particularly of respiratory and abdominal organs, with risk levels of similar magnitude to those mandating urgent referral for suspected cancer investigations.

## Funding

This research aligns with the RREDDEHR project supported by the International Alliance for Cancer Early Detection, a partnership between Cancer Research UK (grant <number>[[ C18081/A31373 to GL), Canary Center at Stanford University, the University of Cambridge, OHSU Knight Cancer Institute, University College London, and the University of Manchester. RP is supported by the University College London British Heart Foundation Research Accelerator (grant <number>[[ AA/18/6/34223), National Institute of Health Research (grant <number>[[ NIHR129463) and UK Research and Innovation European Research Council (10103153 ARISTOTELES). The funders had no role in study design, data collection and analysis, decision to publish, or preparation of the manuscript.

## Data sharing statement

Data used in this study were accessed through NHS Digital that is subject to protocol approval and cannot directly be shared. Requests to access these datasets should be directed to Medicines Healthcare products Regulatory Agency - Clinical Practice Research Datalink, https://cprd.com. Analysis code can be accessed online at https://github.com/nadine-zakkak/cancer-risk-after-atrial-fibrillation.

## CRediT authorship contribution statement

**Nadine Zakkak:** Conceptualization, Data curation, Formal analysis, Methodology, Software, Visualization, Writing – original draft. **Matthew Barclay:** Methodology, Software, Writing – review & editing. **Arturo Gonzalez-Izquierdo:** Software, Writing – review & editing. **Amand Floriaan Schmidt:** Data curation, Formal analysis, Methodology, Software, Writing – review & editing. **Gregory Y.H. Lip:** Conceptualization, Writing – review & editing. **Georgios Lyratzopoulos:** Conceptualization, Resources, Writing – review & editing. **Rui Providencia:** Conceptualization, Methodology, Resources, Writing – review & editing.

## Declaration of competing interest

The authors declare the following financial interests/personal relationships which may be considered as potential competing interests:

**GYHL:** Consultant and speaker for BMS/Pfizer, Boehringer Ingelheim, Daiichi-Sankyo, Anthos. No fees are received personally. GYHL is a National Institute for Health and Care Research (NIHR) Senior Investigator and co-principal investigator of the AFFIRMO project on multimorbidity in AF, which has received funding from the European Union's Horizon 2020 research and innovation programme under grant agreement No 899871.

## Appendix Supplementary materials

Image, application 1

Image, application 2
