# Supplementary material for: Cancer incidence and mortality among patients with new-onset atrial fibrillation: A population-based matched cohort study
Source: Neoplasia. 2024 Nov 8;59:101080. doi: 10.1016/j.neo.2024.101080 (PMC11584679; doi:10.1016/j.neo.2024.101080)
Supplement: Supplementary file 1 [file mmc1.doc]

**STROBE Statement—Checklist of items that should be included in reports of *cohort studies***

|  | Item No | Recommendation | Page No./*Text from manuscript* |
| --- | --- | --- | --- |
| **Title and abstract** | 1 | (*a*) Indicate the study’s design with a commonly used term in the title or the abstract | The title indicates the study’s design: *a population-based matched cohort study* |
|  |  | (*b*) Provide in the abstract an informative and balanced summary of what was done and what was found | The abstract includes data used, main analysis methods and results with a concise conclusion.  ***Methods:*** *Linked electronic health records were used to identify patients aged ≥18 with new-onset AF and age-sex-matched controls. Cumulative incidence of and mortality from cancer (overall and cancer-site specific) within three months, three months to five years and beyond five years from diagnosis of AF were examined.* *Findings were further validated using Mendelian randomisation (MR).*  ***Results:*** *The cohort included 117,173 patients with new-onset AF and 117,173 matched controls (median age 78). In the first three months, 2.2% of AF patients were diagnosed with cancer vs. 0.47% in controls (relative risk: 4.7 [95%CI 4.2-5.4] in men and 4.4 [95%CI 3.8-5.0] in women). Nearly 80% of cancers related to thoracic or abdominal organs. Differences in cumulative incidence were only evident in women between three months and five years (subdistribution hazard ratio=1.1 [95%CI 1.01-1.12]) and absent in all patients beyond five years. MR analysis did not support the presence of a causal association between AF and major cancer subtypes.*  ***Conclusion:*** *There is a large short-term increase in cancer incidence and mortality following new-onset AF. The findings may reflect incidental identification of AF or paraneoplastic manifestation. New-onset AF confers high short-term risk of cancer diagnosis, at levels comparable with symptomatic risk threshold mandating urgent assessment for suspected cancer.* |
| Introduction | | |  |
| Background/rationale | 2 | Explain the scientific background and rationale for the investigation being reported | The introduction explains the importance of examining the association between new-onset atrial fibrillation and subsequent cancer diagnosis and provides context within prior literature. |
| Objectives | 3 | State specific objectives, including any prespecified hypotheses | The final paragraph of the introduction clearly states the study objectives: *This study aims to elucidate whether there is temporal association between new-onset AF and cancer by characterising the risk of mortality from cancer and incidence of cancer overall, and of specific types of cancer across a follow-up time split into three time periods.* |
| Methods | | |  |
| Study design | 4 | Present key elements of study design early in the paper | The beginning the method section clearly defines the study design in the ‘Study Design’ section in the Methods.  ***I. Risk of cancer*** *[…]*  *Study Design  We conducted a population-based retrospective cohort study of patients with new-onset AF and age-sex-matched controls without AF at the start of their follow-up.*  *[…]* |
| Setting | 5 | Describe the setting, locations, and relevant dates, including periods of recruitment, exposure, follow-up, and data collection | Please refer to the sections of the ‘Data and study population’ and the ‘Analysis cohort’  **Data and Study Population**  *Data on 6,529,382 patients in England were provided by the Clinical Practice Research Datalink (CPRD) Gold between 1st January 1998 and 31st May 2016 linked to the national cancer registry, hospital data (Hospital Episode Statistics, HES) and death registry data from the Office for National Statistics (ONS).*  ***Analysis cohort***  *The cases comprised patients aged 18 to 100 years presenting with new-onset AF in primary or secondary care between 1st January 1998 and 31st December 2014. […]  controls comprised patients aged 18 to 101 years with their start of follow-up defined as […]* |
| Participants | 6 | (*a*) Give the eligibility criteria, and the sources and methods of selection of participants. Describe methods of follow-up | Please refer to the sections of the ‘Data and study population’ and the ‘Analysis cohort’. These are also visualised in Figure 1 in a cohort flowchart. |
|  |  | (*b*) For matched studies, give matching criteria and number of exposed and unexposed | The matching criteria is explained in the final paragraph of the ‘Analysis cohort’ section.  *Cases were 1-1 matched with controls based on sex, year of birth (allowing for a 1-year difference) and follow-up period (AF diagnosis occurs within control’s follow-up period). The date of AF diagnosis of the matched case was used as the index date for their corresponding control.* |
| Variables | 7 | Clearly define all outcomes, exposures, predictors, potential confounders, and effect modifiers. Give diagnostic criteria, if applicable | Please refer to the sections of the ‘Study Design’, ‘Covariates’ and ‘Outcomes’  ***Study Design***  *[…] AF was identified in CPRD using Read Codes […]*  ***Covariates***  *We considered age, sex, smoking status, alcohol consumption status, diabetes and hypertension to be potential risk factors for both AF and cancer and adjusted for all or some of the covariates in our analyses. […]*  ***Outcomes***  *Our two primary outcomes were (1) cancer incidence identified from the national cancer registry and (2) cancer mortality identified from the ONS. Secondary outcomes included specific cancer incidence by 8 body regions (abdomen, brain/central nervous system, chest, head and neck, pelvis, upper and lower limbs, unknown primary and other cancers) and 12 organ systems […]* |
| Data sources/ measurement | 8* | For each variable of interest, give sources of data and details of methods of assessment (measurement). Describe comparability of assessment methods if there is more than one group | Please refer to the sections of the ‘Study Design’, ‘Covariates’ and ‘Outcomes’ and supplementary material (Supplemental text S1-S4 and Supplemental tables S1-S5) for clear definitions and sources of data for exposures, covariates and outcomes, |
| Bias | 9 | Describe any efforts to address potential sources of bias | The time period for studying the association between AF and cancer has been split into 3: short term (<= 3 months), medium term (3 months – 5 years) and long term (> 5 years).  ***Analysis***  *We examined associations between new-onset AF and cancer in the short-term (up to 3 months follow-up), medium-term (3 months to 5 years) and long-term (beyond 5 years). These splits were necessary due to evidence of violation of the proportional sub-distribution hazards assumption and allowed us to assess changes in associations over follow-up. All outcomes were examined in each follow-up period.*  *For short-term follow-up analysis, sex-stratified Poisson regression was used, with robust standard errors. This was reasonable as few patients were censored within 3 months. For medium and long follow-up analysis, sex-stratified Fine-Gray regression analysis[15] was used to account for censoring and competing risks (i.e. all-cause death and other cause death for the cancer incidence and cancer mortality outcomes, respectively). […]* |
| Study size | 10 | Explain how the study size was arrived at | This was a retrospective study, the study size was derived from the available data based on the eligibility criteria we have defined, which is clearly stated in the Methods section and Figure 1. (Please refer to the response of point 6 in the checklist). |
| Quantitative variables | 11 | Explain how quantitative variables were handled in the analyses. If applicable, describe which groupings were chosen and why | Age was the only quantitative variable in the analyses and was defined in a continuous format.  Alcohol drinking status was defined in a categorical manner (Current, Ex, Missing or None) as it was not possible to get the exact quantity and duration of alcohol consumption from the data. This limitation is mentioned in the discussion, and the categories used are defined in Supplemental text S4 and Supplemental table S4. |
| Statistical methods | 12 | (*a*) Describe all statistical methods, including those used to control for confounding | Defined in the analysis section:  *[…] For short-term follow-up analysis, sex-stratified Poisson regression was used, with robust standard errors. This was reasonable as few patients were censored within 3 months. For medium and long follow-up analysis, sex-stratified Fine-Gray regression analysis[15] was used to account for censoring and competing risks (i.e. all-cause death and other cause death for the cancer incidence and cancer mortality outcomes, respectively). All models were adjusted for age, diabetes, hypertension, and smoking status. Models with cancer of the digestive system as outcome were additionally adjusted for alcohol consumption status. […]* |
|  |  | (*b*) Describe any methods used to examine subgroups and interactions | Analysis was stratified by sex.  *[…]*  *For short-term follow-up analysis, sex-stratified Poisson regression was used, with robust standard errors. This was reasonable as few patients were censored within 3 months. For medium and long follow-up analysis, sex-stratified Fine-Gray regression analysis[15] was used to account for censoring and competing risks […].* |
|  |  | (*c*) Explain how missing data were addressed | We treated missing data in alcohol drinking status as a separate category and included it in the model (Please refer to Supplemental text S4 and Supplemental table S4). |
|  |  | (*d*) If applicable, explain how loss to follow-up was addressed | It was addressed by censoring the patients who were lost to follow-up and using Fine-Gray regression to analyse the data. |
|  |  | (*e*) Describe any sensitivity analyses | None. |
| Results | | |  |
| Participants | 13* | (a) Report numbers of individuals at each stage of study—eg numbers potentially eligible, examined for eligibility, confirmed eligible, included in the study, completing follow-up, and analysed | The sample size of the cohort used in the analysis is reported in the ‘Cohort description’ section in the Results, with the detailed number of individuals reported at each stage of the cohort derivation process fully displayed in Figure 1.  *The analysis cohort comprised 117,173 patients with new-onset AF and 117,173 matched controls (Fig 1). […]* |
|  |  | (b) Give reasons for non-participation at each stage | Figure 1 explains each step for which patients were excluded from the study. |
|  |  | (c) Consider use of a flow diagram | Please refer to Figure 1. |
| Descriptive data | 14* | (a) Give characteristics of study participants (eg demographic, clinical, social) and information on exposures and potential confounders | The characteristics of the cohort is described in ‘Cohort description’, and Table 1.  *[...] Half (50.6%) of patients were men with a median age of 75 years (IQR 66-82) at index; women were older with a median age of 81 years (IQR 74-87) (Table 1). […]* |
|  |  | (b) Indicate number of participants with missing data for each variable of interest | Table 1 includes the number of participants with missing data for alcohol consumption status. |
|  |  | (c) Summarise follow-up time (eg, average and total amount) | It is summarised in ‘Cohort description’ section and Table 2.  *[…] Patients without AF had longer follow-up periods compared to patients with AF (Table 2). In the cancer mortality analysis, across all outcomes (i.e., cancer death, other cause death, censoring), men with AF were followed-up for a median of 4.1 years vs 5.4 years without AF, while women had shorter follow-up periods (3.4 years with AF vs 5.0 years without AF). […]* |
| Outcome data | 15* | Report numbers of outcome events or summary measures over time | The number of outcome events is detailed in paragraph 2 in the ‘Cohort description’ section, in Table 2 and displayed as a cumulative incidence % over time in Figures 2 and 3.  *Following new-onset of AF, 13,340 patients were diagnosed with cancer: 2528 in the first 3 months, 7184 after 3 months and less than 5 years, and 3628 after the initial 5 years (Table 2). When accounting for censoring, cumulative incidence of cancer was 7.9% at 5 years and 12.7% at 10 years (Supplemental figure S1). Cancer deaths and cancer incidence appeared higher in patients with AF compared to patients without AF in both men and women up to 5 years following the index date (Fig 2). The incidence of cancer by all studied anatomical regions and organ systems was similar in patients with and without AF beyond 3 months of follow-up, or slightly lower, such as cancer in the chest after 10 years of follow-up (Fig 3).* |
| Main results | 16 | (*a*) Give unadjusted estimates and, if applicable, confounder-adjusted estimates and their precision (eg, 95% confidence interval). Make clear which confounders were adjusted for and why they were included | All estimates and their 95% confidence intervals are described in ‘Short-term’, ‘Medium-term’ and ‘Long-term’ sections, and in Figure 4, Supplemental tables S6-7. A clear description of which confounders were used are in the caption of Figure 4, and in the Methods section (Please refer to point 12 of this document).   An example of the results in text:  *[...]*  *2.4% [95% CI 2.28-2.53] of men with AF were diagnosed with cancer within 3 months of their AF diagnosis, compared to 0.5% [95% CI 0.44-0.55] of matched controls. Adjusted risk ratios for cancer death and cancer incidence (overall and cancer-site specific) were higher in patients with new-onset AF compared to their controls in the first 3 months following diagnosis/index (Fig 4; Supplemental table S7). For example, for men, the risk of cancer death was ten times higher in cases than controls (risk ratio (RR) 9.7, [95% CI 7.3-13.0]), while risk of cancer incidence was five times higher (RR 4.7, [95% CI 4.1-5.4]); results for women were broadly similar.*  *[…]* |
|  |  | (*b*) Report category boundaries when continuous variables were categorized | N/A |
|  |  | (*c*) If relevant, consider translating estimates of relative risk into absolute risk for a meaningful time period | N/A |
| Other analyses | 17 | Report other analyses done—eg analyses of subgroups and interactions, and sensitivity analyses | N/A |
| Discussion | | |  |
| Key results | 18 | Summarise key results with reference to study objectives | Please refer to the ‘Summary’ section of the Discussion, where the main results are summarised.  *In this study, we report the cumulative incidence of cancer, overall and of specific types, and of cancer death in three time-periods following AF diagnosis: initial 3 months, 3 months up to 5 years and beyond 5 years. The vast majority of cancers diagnosed following an AF presentation were located in the chest, abdomen, or pelvis.*  *Our principal findings are as follows: (i) Risk of cancer was high immediately following a new diagnosis of AF but rapidly became similar to the risk of cancer in controls. Compared to their controls, patients with incident AF had a higher risk of cancer following AF diagnosis within the first 3 months in both men and women, and between 3 months and 5 years in women, without a difference in the risk of cancer in patients with and without AF between 3 months and 5 years in men and beyond 5 years in both men and women. Our Mendelian Randomisation analyses does not support the presence of a causal association between AF and lung, breast or colorectal cancer; and (ii) The cumulative incidence of cancer death remained increased in men with AF beyond 5 years following diagnosis compared to patients without AF, while the cumulative incidence was increased in women up to 5 years following AF and there was no difference in risk beyond 5 years.* |
| Limitations | 19 | Discuss limitations of the study, taking into account sources of potential bias or imprecision. Discuss both direction and magnitude of any potential bias | This is discussed in the ‘Strengths and limitations’ section:  *[…]*  *Using electronic health records, we were able to obtain a large sample size of AF cases and their matched controls. However, some AF cases might have been misclassified due to coding, despite our use of a validated AF phenotype.[26] Accounting for more detailed alcohol and smoking statuses is likely to improve the models because the quantity and duration of alcoholic drinking and smoking might change the risks of AF and cancer; however, these are difficult to capture in electronic health records.[12] As for any observational study, there is always a risk of unmeasured confounders (e.g. potentially carcinogenic agents that did not occur at random across AF patients and controls).* |
| Interpretation | 20 | Give a cautious overall interpretation of results considering objectives, limitations, multiplicity of analyses, results from similar studies, and other relevant evidence | An interpretation of the results and comparison to similar to studies have been discussed in the sections ‘Comparison with literature’ and ‘Interpretation and clinical relevance’.  Please see an example text from the mentioned sections below: Comparison with literature *[…]*  *Similar to previous studies, we found an increased risk of cancer following an incident AF diagnosis that weakened over time.[6,8,9] The highest risk of cancer appeared to be within the first 3 months following AF diagnosis, with two studies reporting an increased risk up to 1 year after AF diagnosis,[6,9] while we have found a slight increase in risk up to 5 years following AF in women and no difference in risk in men beyond 3 months. In concordance with a previous study, the Mendelian Randomisation analyses did not confirm a causal association between AF and lung, breast or colorectal cancer.[23]*  *[…]* Interpretation and clinical relevance *Our study is not equipped to provide complete aetiological proof of the observed association. However, we can consider three principal potential mechanisms, comprising two non-causal and one causal explanation. First, the increased risk of cancer, overall and all studied types, within the first 3 months after AF diagnosis that decreases and disappears over time suggests the possibility of incidental identification of asymptomatic cancer after presentation with AF symptoms. Such incidental identification is unlikely to be dominant given the strong association with short-term cancer mortality; incidentally diagnosed cancers are typically early stage and have good short-term prognosis. Second, […]* |
| Generalisability | 21 | Discuss the generalisability (external validity) of the study results | This is discussed in the ‘Strengths and limitations’ section:  *[…] Data from CPRD have high completeness of clinical information recorded [26, 27], and are generally representative of the age, gender and geographic distribution of the UK population.[28] While the data relate to UK patients, there are no comparable population-based data sources incorporating information from primary care and hospital data in other countries, nonetheless the disease associations we describe are likely to be overall generalisable to a great degree.* |
| Other information | | |  |
| Funding | 22 | Give the source of funding and the role of the funders for the present study and, if applicable, for the original study on which the present article is based | Please refer to the ‘Funding’ section  ***Funding***  *This research aligns with the RREDDEHR project supported by the International Alliance for Cancer Early Detection (C18081/A31373), a partnership between Cancer Research UK (grant number C18081/A31373 to GL), Canary Center at Stanford University, the University of Cambridge, OHSU Knight Cancer Institute, University College London, and the University of Manchester. RP is supported by the University College London British Heart Foundation Research Accelerator (grant number AA/18/6/34223) and National Institute of Health Research (grant number NIHR129463).*  *The funders had no role in study design, data collection and analysis, decision to publish, or preparation of the manuscript.* |

*Give information separately for exposed and unexposed groups.

**Note:** An Explanation and Elaboration article discusses each checklist item and gives methodological background and published examples of transparent reporting. The STROBE checklist is best used in conjunction with this article (freely available on the Web sites of PLoS Medicine at http://www.plosmedicine.org/, Annals of Internal Medicine at http://www.annals.org/, and Epidemiology at http://www.epidem.com/). Information on the STROBE Initiative is available at http://www.strobe-statement.org.
