# Supplementary material for: Cancer incidence and mortality among patients with new-onset atrial fibrillation: A population-based matched cohort study
Source: Neoplasia. 2024 Nov 8;59:101080. doi: 10.1016/j.neo.2024.101080 (PMC11584679; doi:10.1016/j.neo.2024.101080)
Supplement: Supplementary file 2 [file mmc2.docx]

Supplementary material

## Table of contents

[Text S1. Definition of patients with diabetes. 2](#_Toc170903394)

[Text S2. Definition of patients with hypertension. 8](#_Toc170903395)

[Text S3. Definition of the smoking status of patients. 10](#_Toc170903396)

[Text S4. Definition of the alcohol drinking status of patients. 12](#_Toc170903397)

[Table S1. Read codes to identify patients with diabetes. 2](#_Toc170903399)

[Table S2. Read codes to identify patients with hypertension. 8](#_Toc170903400)

[Table S3. Read codes to identify patients that are current or ex- smokers. 10](#_Toc170903401)

[Table S4. Read codes to identify alcohol drinking status of patients. 12](#_Toc170903402)

[Table S5. ICD-10 codes of cancer outcomes and categorisation into anatomical regions and organ systems. 13](#_Toc170903403)

[Table S6. Proportion of outcomes at different time points for patients with new-onset atrial fibrillation (AF) and their matched controls, split by men and women. 19](#_Toc170903404)

[Table S7. Adjusted model estimates of all outcomes. 23](#_Toc170903405)

[Table S8. Mendelian Randomisation analyses estimates. 28](#_Toc170903406)

[Figure S1. Incidence of cancer after new-onset atrial fibrillation. 18](#_Toc170903407)

[Figure S2. Distribution of cancer sites by body region in patients with new-onset atrial fibrillation that were diagnosed with cancer within 3 months. 27](#_Toc170903408)

[Figure S3. Distribution of cancer sites by organ system in patients with new-onset atrial fibrillation that were diagnosed with cancer within 3 months. 27](#_Toc170903409)

Text S1. Definition of patients with diabetes.

| A patient was listed as a diabetic if any of the following ICD-10 codes (E10, E11, E13, E14, G590, G632, H280, H360, M142, N083, O240, O241, O243) or read codes in **Supplementary table S1** were present in their records up to the index date.  Phenotypes from CALIBER can be accessed at <https://phenotypes.healthdatagateway.org/phenotypes/?collection_ids=21> |
| --- |

Table S1. Read codes to identify patients with diabetes.

| Read Term | | Read Code | |
| --- | --- | --- | --- |
| Diabetes type 1 review | | 66An.00 | |
| Diabetes mellitus, juvenile type, no mention of complication | | C100000 | |
| Insulin dependent diabetes mellitus | | C100011 | |
| Diabetes mellitus, juvenile type, with ketoacidosis | | C101000 | |
| Diabetes mellitus, juvenile type, with hyperosmolar coma | | C102000 | |
| Diabetes mellitus, juvenile type, with ketoacidotic coma | | C103000 | |
| Diabetes mellitus, juvenile type, with renal manifestation | | C104000 | |
| Diabetes mellitus, juvenile type, + ophthalmic manifestation | | C105000 | |
| Diabetes mellitus, juvenile, + neurological manifestation | | C106000 | |
| Diabetes mellitus, juvenile +peripheral circulatory disorder | | C107000 | |
| IDDM with peripheral circulatory disorder | | C107300 | |
| Insulin dependent diabetes mellitus | | C108.00 | |
| IDDM-Insulin dependent diabetes mellitus | | C108.11 | |
| Type 1 diabetes mellitus | | C108.12 | |
| Type I diabetes mellitus | | C108.13 | |
| Insulin-dependent diabetes mellitus with renal complications | | C108000 | |
| Type I diabetes mellitus with renal complications | | C108011 | |
| Type 1 diabetes mellitus with renal complications | | C108012 | |
| Insulin-dependent diabetes mellitus with ophthalmic comps | | C108100 | |
| Insulin-dependent diabetes mellitus with neurological comps | | C108200 | |
| Type I diabetes mellitus with neurological complications | | C108211 | |
| Type 1 diabetes mellitus with neurological complications | | C108212 | |
| Insulin dependent diabetes mellitus with multiple complicatn | | C108300 | |
| Unstable insulin dependant diabetes mellitus | | C108400 | |
| Unstable type I diabetes mellitus | | C108411 | |
| Unstable type 1 diabetes mellitus | | C108412 | |
| Insulin dependent diabetes mellitus with ulcer | | C108500 | |
| Type I diabetes mellitus with ulcer | | C108511 | |
| Type 1 diabetes mellitus with ulcer | | C108512 | |
| Insulin dependent diabetes mellitus with gangrene | | C108600 | |
| Insulin dependent diabetes mellitus with retinopathy | | C108700 | |
| Type I diabetes mellitus with retinopathy | | C108711 | |
| Type 1 diabetes mellitus with retinopathy | | C108712 | |
| Insulin dependant diabetes mellitus - poor control | | C108800 | |
| Type I diabetes mellitus - poor control | | C108811 | |
| Type 1 diabetes mellitus - poor control | | C108812 | |
| Insulin dependant diabetes maturity onset | | C108900 | |
| Type I diabetes mellitus maturity onset | | C108911 | |
| Type 1 diabetes mellitus maturity onset | | C108912 | |
| Insulin-dependent diabetes without complication | | C108A00 | |
| Type I diabetes mellitus without complication | | C108A11 | |
| Insulin dependent diabetes mellitus with mononeuropathy | | C108B00 | |
| Insulin dependent diabetes mellitus with polyneuropathy | | C108C00 | |
| Insulin dependent diabetes mellitus with nephropathy | | C108D00 | |
| Type I diabetes mellitus with nephropathy | | C108D11 | |
| Insulin dependent diabetes mellitus with hypoglycaemic coma | | C108E00 | |
| Type I diabetes mellitus with hypoglycaemic coma | | C108E11 | |
| Type 1 diabetes mellitus with hypoglycaemic coma | | C108E12 | |
| Insulin dependent diabetes mellitus with diabetic cataract | | C108F00 | |
| Type I diabetes mellitus with diabetic cataract | | C108F11 | |
| Insulin dependent diab mell with peripheral angiopathy | | C108G00 | |
| Insulin dependent diabetes mellitus with arthropathy | | C108H00 | |
| Type I diabetes mellitus with arthropathy | | C108H11 | |
| Insulin dependent diab mell with neuropathic arthropathy | | C108J00 | |
| Type I diabetes mellitus with neuropathic arthropathy | | C108J11 | |
| Type 1 diabetes mellitus with neuropathic arthropathy | | C108J12 | |
| Maturity onset diabetes in youth type 1 | | C10C.12 | |
| Type 1 diabetes mellitus | | C10E.00 | |
| Type I diabetes mellitus | | C10E.11 | |
| Insulin dependent diabetes mellitus | | C10E.12 | |
| Type 1 diabetes mellitus with renal complications | | C10E000 | |
| Type 1 diabetes mellitus with ophthalmic complications | | C10E100 | |
| Insulin-dependent diabetes mellitus with ophthalmic comps | | C10E112 | |
| Type 1 diabetes mellitus with neurological complications | | C10E200 | |
| Type 1 diabetes mellitus with multiple complications | | C10E300 | |
| Type I diabetes mellitus with multiple complications | | C10E311 | |
| Insulin dependent diabetes mellitus with multiple complicat | | C10E312 | |
| Unstable type 1 diabetes mellitus | | C10E400 | |
| Unstable type I diabetes mellitus | | C10E411 | |
| Unstable insulin dependent diabetes mellitus | | C10E412 | |
| Type 1 diabetes mellitus with ulcer | | C10E500 | |
| Type I diabetes mellitus with ulcer | | C10E511 | |
| Insulin dependent diabetes mellitus with ulcer | | C10E512 | |
| Type 1 diabetes mellitus with gangrene | | C10E600 | |
| Type 1 diabetes mellitus with retinopathy | | C10E700 | |
| Type I diabetes mellitus with retinopathy | | C10E711 | |
| Insulin dependent diabetes mellitus with retinopathy | | C10E712 | |
| Type 1 diabetes mellitus - poor control | | C10E800 | |
| Insulin dependent diabetes mellitus - poor control | | C10E812 | |
| Type 1 diabetes mellitus maturity onset | | C10E900 | |
| Type I diabetes mellitus maturity onset | | C10E911 | |
| Insulin dependent diabetes maturity onset | | C10E912 | |
| Type 1 diabetes mellitus without complication | | C10EA00 | |
| Type I diabetes mellitus without complication | | C10EA11 | |
| Type 1 diabetes mellitus with mononeuropathy | | C10EB00 | |
| Type 1 diabetes mellitus with polyneuropathy | | C10EC00 | |
| Type I diabetes mellitus with polyneuropathy | | C10EC11 | |
| Type 1 diabetes mellitus with nephropathy | | C10ED00 | |
| Type 1 diabetes mellitus with hypoglycaemic coma | | C10EE00 | |
| Type 1 diabetes mellitus with diabetic cataract | | C10EF00 | |
| Type 1 diabetes mellitus with peripheral angiopathy | | C10EG00 | |
| Type 1 diabetes mellitus with arthropathy | | C10EH00 | |
| Type 1 diabetes mellitus with neuropathic arthropathy | | C10EJ00 | |
| Type 1 diabetes mellitus with persistent proteinuria | | C10EK00 | |
| Type 1 diabetes mellitus with persistent microalbuminuria | | C10EL00 | |
| Type 1 diabetes mellitus with ketoacidosis | | C10EM00 | |
| Type I diabetes mellitus with ketoacidosis | | C10EM11 | |
| Type 1 diabetes mellitus with ketoacidotic coma | | C10EN00 | |
| Type I diabetes mellitus with ketoacidotic coma | | C10EN11 | |
| Type 1 diabetes mellitus with exudative maculopathy | | C10EP00 | |
| Type I diabetes mellitus with exudative maculopathy | | C10EP11 | |
| Type 1 diabetes mellitus with gastroparesis | | C10EQ00 | |
| Diabetes mellitus, juvenile type, + unspecified complication | | C10z000 | |
| Pre-existing diabetes mellitus, insulin-dependent | | L180500 | |
| Insulin lipohypertrophy | | M21yC00 | |
| Insulin site lipohypertrophy | | M21yC11 | |
| Dietary advice for type I diabetes | | ZC2C900 | |
| Perceived control of insulin-dependent diabetes | | ZRbH.00 | |
| Diabetes type 2 review | | 66Ao.00 | |
| Diabetes mellitus, adult onset, no mention of complication | | C100100 | |
| Maturity onset diabetes | | C100111 | |
| Non-insulin dependent diabetes mellitus | | C100112 | |
| Diabetes mellitus, adult onset, with ketoacidosis | | C101100 | |
| Diabetes mellitus, adult onset, with hyperosmolar coma | | C102100 | |
| Diabetes mellitus, adult onset, with ketoacidotic coma | | C103100 | |
| Diabetes mellitus, adult onset, with renal manifestation | | C104100 | |
| Diabetes mellitus, adult onset, + ophthalmic manifestation | | C105100 | |
| Diabetes mellitus, adult onset, + neurological manifestation | | C106100 | |
| Diabetes mellitus, adult, + peripheral circulatory disorder | | C107100 | |
| Diabetes mellitus, adult with gangrene | | C107200 | |
| NIDDM with peripheral circulatory disorder | | C107400 | |
| Non-insulin dependent diabetes mellitus | | C109.00 | |
| NIDDM - Non-insulin dependent diabetes mellitus | | C109.11 | |
| Type 2 diabetes mellitus | | C109.12 | |
| Type II diabetes mellitus | | C109.13 | |
| Non-insulin-dependent diabetes mellitus with renal comps | | C109000 | |
| Type II diabetes mellitus with renal complications | | C109011 | |
| Type 2 diabetes mellitus with renal complications | | C109012 | |
| Non-insulin-dependent diabetes mellitus with ophthalm comps | | C109100 | |
| Type II diabetes mellitus with ophthalmic complications | | C109111 | |
| Type 2 diabetes mellitus with ophthalmic complications | | C109112 | |
| Non-insulin-dependent diabetes mellitus with neuro comps | | C109200 | |
| Type II diabetes mellitus with neurological complications | | C109211 | |
| Type 2 diabetes mellitus with neurological complications | | C109212 | |
| Non-insulin-dependent diabetes mellitus with multiple comps | | C109300 | |
| Non-insulin dependent diabetes mellitus with ulcer | | C109400 | |
| Type II diabetes mellitus with ulcer | | C109411 | |
| Type 2 diabetes mellitus with ulcer | | C109412 | |
| Non-insulin dependent diabetes mellitus with gangrene | | C109500 | |
| Type II diabetes mellitus with gangrene | | C109511 | |
| Type 2 diabetes mellitus with gangrene | | C109512 | |
| Non-insulin-dependent diabetes mellitus with retinopathy | | C109600 | |
| Type II diabetes mellitus with retinopathy | | C109611 | |
| Type 2 diabetes mellitus with retinopathy | | C109612 | |
| Non-insulin dependant diabetes mellitus - poor control | | C109700 | |
| Type II diabetes mellitus - poor control | | C109711 | |
| Type 2 diabetes mellitus - poor control | | C109712 | |
| Non-insulin-dependent diabetes mellitus without complication | | C109900 | |
| Non-insulin dependent diabetes mellitus with mononeuropathy | | C109A00 | |
| Type II diabetes mellitus with mononeuropathy | | C109A11 | |
| Non-insulin dependent diabetes mellitus with polyneuropathy | | C109B00 | |
| Type II diabetes mellitus with polyneuropathy | | C109B11 | |
| Non-insulin dependent diabetes mellitus with nephropathy | | C109C00 | |
| Type II diabetes mellitus with nephropathy | | C109C11 | |
| Type 2 diabetes mellitus with nephropathy | | C109C12 | |
| Non-insulin dependent diabetes mellitus with hypoglyca coma | | C109D00 | |
| Type II diabetes mellitus with hypoglycaemic coma | | C109D11 | |
| Type 2 diabetes mellitus with hypoglycaemic coma | | C109D12 | |
| Non-insulin depend diabetes mellitus with diabetic cataract | | C109E00 | |
| Type II diabetes mellitus with diabetic cataract | | C109E11 | |
| Type 2 diabetes mellitus with diabetic cataract | | C109E12 | |
| Non-insulin-dependent d m with peripheral angiopath | | C109F00 | |
| Type II diabetes mellitus with peripheral angiopathy | | C109F11 | |
| Type 2 diabetes mellitus with peripheral angiopathy | | C109F12 | |
| Non-insulin dependent diabetes mellitus with arthropathy | | C109G00 | |
| Type II diabetes mellitus with arthropathy | | C109G11 | |
| Type 2 diabetes mellitus with arthropathy | | C109G12 | |
| Non-insulin dependent d m with neuropathic arthropathy | | C109H00 | |
| Type II diabetes mellitus with neuropathic arthropathy | | C109H11 | |
| Type 2 diabetes mellitus with neuropathic arthropathy | | C109H12 | |
| Insulin treated Type 2 diabetes mellitus | | C109J00 | |
| Insulin treated non-insulin dependent diabetes mellitus | | C109J11 | |
| Insulin treated Type II diabetes mellitus | | C109J12 | |
| Hyperosmolar non-ketotic state in type 2 diabetes mellitus | | C109K00 | |
| Maturity onset diabetes in youth | | C10C.11 | |
| Diabetes mellitus autosomal dominant type 2 | | C10D.00 | |
| Maturity onset diabetes in youth type 2 | | C10D.11 | |
| Latent autoimmune diabetes mellitus in adult | | C10ER00 | |
| Type 2 diabetes mellitus | | C10F.00 | |
| Type II diabetes mellitus | | C10F.11 | |
| Type 2 diabetes mellitus with renal complications | | C10F000 | |
| Type II diabetes mellitus with renal complications | | C10F011 | |
| Type 2 diabetes mellitus with ophthalmic complications | | C10F100 | |
| Type 2 diabetes mellitus with neurological complications | | C10F200 | |
| Type II diabetes mellitus with neurological complications | | C10F211 | |
| Type 2 diabetes mellitus with multiple complications | | C10F300 | |
| Type II diabetes mellitus with multiple complications | | C10F311 | |
| Type 2 diabetes mellitus with ulcer | | C10F400 | |
| Type II diabetes mellitus with ulcer | | C10F411 | |
| Type 2 diabetes mellitus with gangrene | | C10F500 | |
| Type 2 diabetes mellitus with retinopathy | | C10F600 | |
| Type II diabetes mellitus with retinopathy | | C10F611 | |
| Type 2 diabetes mellitus - poor control | | C10F700 | |
| Type II diabetes mellitus - poor control | | C10F711 | |
| Type 2 diabetes mellitus without complication | | C10F900 | |
| Type II diabetes mellitus without complication | | C10F911 | |
| Type 2 diabetes mellitus with mononeuropathy | | C10FA00 | |
| Type II diabetes mellitus with mononeuropathy | | C10FA11 | |
| Type 2 diabetes mellitus with polyneuropathy | | C10FB00 | |
| Type II diabetes mellitus with polyneuropathy | | C10FB11 | |
| Type 2 diabetes mellitus with nephropathy | | C10FC00 | |
| Type 2 diabetes mellitus with hypoglycaemic coma | | C10FD00 | |
| Type II diabetes mellitus with hypoglycaemic coma | | C10FD11 | |
| Type 2 diabetes mellitus with diabetic cataract | | C10FE00 | |
| Type II diabetes mellitus with diabetic cataract | | C10FE11 | |
| Type 2 diabetes mellitus with peripheral angiopathy | | C10FF00 | |
| Type 2 diabetes mellitus with arthropathy | | C10FG00 | |
| Type 2 diabetes mellitus with neuropathic arthropathy | | C10FH00 | |
| Insulin treated Type 2 diabetes mellitus | | C10FJ00 | |
| Insulin treated Type II diabetes mellitus | | C10FJ11 | |
| Hyperosmolar non-ketotic state in type 2 diabetes mellitus | | C10FK00 | |
| Type 2 diabetes mellitus with persistent proteinuria | | C10FL00 | |
| Type II diabetes mellitus with persistent proteinuria | | C10FL11 | |
| Type 2 diabetes mellitus with persistent microalbuminuria | | C10FM00 | |
| Type II diabetes mellitus with persistent microalbuminuria | | C10FM11 | |
| Type 2 diabetes mellitus with ketoacidosis | | C10FN00 | |
| Type 2 diabetes mellitus with ketoacidotic coma | | C10FP00 | |
| Type 2 diabetes mellitus with exudative maculopathy | | C10FQ00 | |
| Type 2 diabetes mellitus with gastroparesis | | C10FR00 | |
| Diabetes mellitus, adult, + other specified manifestation | | C10y100 | |
| Diabetes mellitus, adult onset, + unspecified complication | | C10z100 | |
| Pre-existing diabetes mellitus, non-insulin-dependent | | L180600 | |
| Dietary advice for type II diabetes | | ZC2CA00 | |
| Diabetic lipid lowering diet | | 13AB.00 | |
| Diabetic weight reducing diet | | 13AC.00 | |
| Diabetic diet | | 13B1.00 | |
| Retinal abnormality - diabetes related | | 2BBF.00 | |
| O/E - diabetic maculopathy present both eyes | | 2BBL.00 | |
| O/E - diabetic maculopathy absent both eyes | | 2BBM.00 | |
| O/E - right eye background diabetic retinopathy | | 2BBP.00 | |
| O/E - left eye background diabetic retinopathy | | 2BBQ.00 | |
| O/E - right eye preproliferative diabetic retinopathy | | 2BBR.00 | |
| O/E - left eye preproliferative diabetic retinopathy | | 2BBS.00 | |
| O/E - right eye proliferative diabetic retinopathy | | 2BBT.00 | |
| O/E - left eye proliferative diabetic retinopathy | | 2BBV.00 | |
| O/E - right eye diabetic maculopathy | | 2BBW.00 | |
| O/E - left eye diabetic maculopathy | | 2BBX.00 | |
| O/E - right eye stable treated prolif diabetic retinopathy | | 2BBk.00 | |
| O/E - left eye stable treated prolif diabetic retinopathy | | 2BBl.00 | |
| O/E - sight threatening diabetic retinopathy | | 2BBo.00 | |
| Foot abnormality - diabetes related | | 2G51000 | |
| O/E - Right diabetic foot at risk | | 2G5A.00 | |
| O/E - Left diabetic foot at risk | | 2G5B.00 | |
| Foot abnormality - diabetes related | | 2G5C.00 | |
| O/E - Right diabetic foot at low risk | | 2G5E.00 | |
| O/E - Right diabetic foot at moderate risk | | 2G5F.00 | |
| O/E - Right diabetic foot at high risk | | 2G5G.00 | |
| O/E - Right diabetic foot - ulcerated | | 2G5H.00 | |
| O/E - Left diabetic foot at low risk | | 2G5I.00 | |
| O/E - Left diabetic foot at moderate risk | | 2G5J.00 | |
| O/E - Left diabetic foot at high risk | | 2G5K.00 | |
| O/E - Left diabetic foot - ulcerated | | 2G5L.00 | |
| O/E - right chronic diabetic foot ulcer | | 2G5V.00 | |
| O/E - left chronic diabetic foot ulcer | | 2G5W.00 | |
| Diabetic on diet only | | 66A3.00 | |
| Diabetic on oral treatment | | 66A4.00 | |
| Diabetic on insulin | | 66A5.00 | |
| Has seen dietician - diabetes | | 66A8.00 | |
| Understands diet - diabetes | | 66A9.00 | |
| Injection sites - diabetic | | 66AA.11 | |
| Fundoscopy - diabetic check | | 66AD.00 | |
| Diabetic drug side effects | | 66AG.00 | |
| Diabetic treatment changed | | 66AH.00 | |
| Diabetic - good control | | 66AI.00 | |
| Diabetic - poor control | | 66AJ.00 | |
| Unstable diabetes | | 66AJ.11 | |
| Brittle diabetes | | 66AJ100 | |
| Diabetic - poor control NOS | | 66AJz00 | |
| Diabetic - cooperative patient | | 66AK.00 | |
| Diabetic-uncooperative patient | | 66AL.00 | |
| Date diabetic treatment start | | 66AN.00 | |
| Date diabetic treatment stopp. | | 66AO.00 | |
| Diabetes: practice programme | | 66AP.00 | |
| Diabetes: shared care programme | | 66AQ.00 | |
| Diabetes management plan given | | 66AR.00 | |
| Diabetic annual review | | 66AS.00 | |
| Annual diabetic blood test | | 66AT.00 | |
| Diabetes care by hospital only | | 66AU.00 | |
| Diabetic on insulin and oral treatment | | 66AV.00 | |
| Diabetic foot risk assessment | | 66AW.00 | |
| Diabetes: shared care in pregnancy - diabetol and obstet | | 66AX.00 | |
| Diabetic diet - good compliance | | 66AY.00 | |
| Diabetic diet - poor compliance | | 66Aa.00 | |
| Diabetic foot examination | | 66Ab.00 | |
| Diabetic peripheral neuropathy screening | | 66Ac.00 | |
| Insulin needles changed daily | | 66Ag.00 | |
| Insulin needles changed for each injection | | 66Ah.00 | |
| Diabetic 6 month review | | 66Ai.00 | |
| Insulin needles changed less than once a day | | 66Aj.00 | |
| Insulin dose changed | | 66Am.00 | |
| Insulin treatment initiated | | 66Ap.00 | |
| Diabetic foot screen | | 66Aq.00 | |
| Diabetic pre-pregnancy counselling | | 6761 | |
| Diabetic retinopathy screening | | 68A7.00 | |
| Diabetic retinopathy screening offered | | 68A9.00 | |
| Diabetic digital retinopathy screening offered | | 68AB.00 | |
| Pan retinal photocoagulation for diabetes | | 7276 | |
| Continuous subcutaneous infusion of insulin | | 7L10000 | |
| Subcutaneous injection of insulin | | 7L19800 | |
| Diab mellit insulin-glucose infus acute myocardial infarct | | 889A.00 | |
| Diabetic stabilisation | | 8A13.00 | |
| Diabetes medication review | | 8B3l.00 | |
| Patient on maximal tolerated therapy for diabetes | | 8BL2.00 | |
| Pt advised re diabetic diet | | 8CA4100 | |
| Advice about blood glucose control | | 8CAQ.00 | |
| Transition of diabetes care options discussed | | 8CP2.00 | |
| Admit diabetic emergency | | 8H2J.00 | |
| Non-urgent diabetic admission | | 8H3O.00 | |
| Refer to diabetic foot screener | | 8H7r.00 | |
| Diabetic retinopathy 12 month review | | 8HBG.00 | |
| Diabetic retinopathy 6 month review | | 8HBH.00 | |
| Diabetology D.V. done | | 8HLE.00 | |
| Referral for diabetic retinopathy screening | | 8Hl1.00 | |
| Diabetic foot examination declined | | 8I3W.00 | |
| Diabetic retinopathy screening refused | | 8I3X.00 | |
| Insulin therapy declined | | 8I3k.00 | |
| Patient held diabetic record declined | | 8I57.00 | |
| Patient held diabetic record issued | | 9360 | |
| Diabetic patient unsuitable for digital retinal photography | | 9OLD.00 | |
| Diabetes mellitus | | C10..00 | |
| Diabetes mellitus with no mention of complication | | C100.00 | |
| Diabetes mellitus NOS with no mention of complication | | C100z00 | |
| Diabetes mellitus with ketoacidosis | | C101.00 | |
| Other specified diabetes mellitus with ketoacidosis | | C101y00 | |
| Diabetes mellitus NOS with ketoacidosis | | C101z00 | |
| Diabetes mellitus with hyperosmolar coma | | C102.00 | |
| Diabetes mellitus NOS with hyperosmolar coma | | C102z00 | |
| Diabetes mellitus with ketoacidotic coma | | C103.00 | |
| Other specified diabetes mellitus with coma | | C103y00 | |
| Diabetes mellitus NOS with ketoacidotic coma | | C103z00 | |
| Diabetes mellitus with renal manifestation | | C104.00 | |
| Diabetic nephropathy | | C104.11 | |
| Other specified diabetes mellitus with renal complications | | C104y00 | |
| Diabetes mellitis with nephropathy NOS | | C104z00 | |
| Diabetes mellitus with ophthalmic manifestation | | C105.00 | |
| Other specified diabetes mellitus with ophthalmic complicatn | | C105y00 | |
| Diabetes mellitus NOS with ophthalmic manifestation | | C105z00 | |
| Diabetes mellitus with neurological manifestation | | C106.00 | |
| Diabetic amyotrophy | | C106.11 | |
| Diabetes mellitus with neuropathy | | C106.12 | |
| Diabetes mellitus with polyneuropathy | | C106.13 | |
| Other specified diabetes mellitus with neurological comps | | C106y00 | |
| Diabetes mellitus NOS with neurological manifestation | | C106z00 | |
| Diabetes mellitus with peripheral circulatory disorder | | C107.00 | |
| Diabetes mellitus with gangrene | | C107.11 | |
| Diabetes with gangrene | | C107.12 | |
| Diabetes mellitus NOS with peripheral circulatory disorder | | C107z00 | |
| Other specified diabetes mellitus with multiple comps | | C108y00 | |
| Unspecified diabetes mellitus with multiple complications | | C108z00 | |
| Malnutrition-related diabetes mellitus | | C10A.00 | |
| Malnutrition-related diabetes mellitus with coma | | C10A000 | |
| Malnutrition-related diabetes mellitus with ketoacidosis | | C10A100 | |
| Diabetes mellitus autosomal dominant | | C10C.00 | |
| Lipoatrophic diabetes mellitus | | C10M.00 | |
| Diabetes mellitus with other specified manifestation | | C10y.00 | |
| Other specified diabetes mellitus with other spec comps | | C10yy00 | |
| Diabetes mellitus NOS with other specified manifestation | | C10yz00 | |
| Diabetes mellitus with unspecified complication | | C10z.00 | |
| Other specified diabetes mellitus with unspecified comps | | C10zy00 | |
| Diabetes mellitus NOS with unspecified complication | | C10zz00 | |
| Renal diabetes | | C314.11 | |
| Bronzed diabetes | | C350011 | |
| [X]Diabetes mellitus | | Cyu2.00 | |
| [X]Other specified diabetes mellitus | | Cyu2000 | |
| Autonomic neuropathy due to diabetes | | F171100 | |
| Diabetic mononeuritis multiplex | | F345000 | |
| Diabetic mononeuritis NOS | | F35z000 | |
| Polyneuropathy in diabetes | | F372.00 | |
| Diabetic polyneuropathy | | F372.11 | |
| Diabetic neuropathy | | F372.12 | |
| Acute painful diabetic neuropathy | | F372000 | |
| Chronic painful diabetic neuropathy | | F372100 | |
| Asymptomatic diabetic neuropathy | | F372200 | |
| Myasthenic syndrome due to diabetic amyotrophy | | F381300 | |
| Diabetic amyotrophy | | F381311 | |
| Diabetic mononeuropathy | | F3y0.00 | |
| Diabetic retinopathy | | F420.00 | |
| Background diabetic retinopathy | | F420000 | |
| Proliferative diabetic retinopathy | | F420100 | |
| Preproliferative diabetic retinopathy | | F420200 | |
| Advanced diabetic maculopathy | | F420300 | |
| Diabetic maculopathy | | F420400 | |
| Advanced diabetic retinal disease | | F420500 | |
| Non proliferative diabetic retinopathy | | F420600 | |
| High risk proliferative diabetic retinopathy | | F420700 | |
| High risk non proliferative diabetic retinopathy | | F420800 | |
| Diabetic retinopathy NOS | | F420z00 | |
| Diabetic iritis | | F440700 | |
| Diabetic cataract | | F464000 | |
| Diabetic peripheral angiopathy | | G73y000 | |
| Nephrotic syndrome in diabetes mellitus | | K01x100 | |
| Kimmelstiel - Wilson disease | | K01x111 | |
| Pre-existing diabetes mellitus, unspecified | | L180X00 | |
| Cellulitis in diabetic foot | | M037200 | |
| Ischaemic ulcer diabetic foot | | M271000 | |
| Neuropathic diabetic ulcer - foot | | M271100 | |
| Mixed diabetic ulcer - foot | | M271200 | |
| Diabetic cheiroarthropathy | | N030000 | |
| Diabetic cheiropathy | | N030011 | |
| Diabetic Charcot arthropathy | | N030100 | |
| Neonatal diabetes mellitus | | Q441.00 | |
| [D]Gangrene of toe in diabetic | | R054200 | |
| [D]Widespread diabetic foot gangrene | | R054300 | |
| Adverse reaction to insulins and antidiabetic agents | | TJ23.00 | |
| Adverse reaction to insulins and antidiabetic agents NOS | | TJ23z00 | |
| [X] Adverse reaction to insulins and antidiabetic agents | | U602311 | |
| Dietary advice for diabetes mellitus | | ZC2C800 | |
| [V]Dietary counselling in diabetes mellitus | ZV65312 | |  |

Text S2. Definition of patients with hypertension.

| A patient was listed as hypertensive if any of the following ICD-10 codes (I10, I11, I12, I13, I15) or read codes in **Supplementary table S2** were present in their records up to the index date. Phenotypes from CALIBER can be accessed at <https://phenotypes.healthdatagateway.org/phenotypes/?collection_ids=21> |
| --- |

Table S2. Read codes to identify patients with hypertension.

| Read Term | Read Code |
| --- | --- |
| Borderline hyperten:yearly obs | 6624 |
| Good hypertension control | 6627 |
| Poor hypertension control | 6628 |
| Hypertension treatm. started | 662F.00 |
| Hypertensive treatm.changed | 662G.00 |
| On treatment for hypertension | 662O.00 |
| Moderate hypertension control | 662b.00 |
| Hypertension six month review | 662c.00 |
| Hypertension annual review | 662d.00 |
| Trial withdrawal of antihypertensive therapy | 662r.00 |
| High cost hypertension drugs | 7Q01.00 |
| Antihypertensive therapy | 8B26.00 |
| Patient on maximal tolerated antihypertensive therapy | 8BL0.00 |
| Hypertension treatment refused | 8I3N.00 |
| Blind hypertensive eye | F404200 |
| Hypertensive retinopathy | F421300 |
| Hypertensive disease | G2...00 |
| BP - hypertensive disease | G2...11 |
| Essential hypertension | G20..00 |
| Malignant essential hypertension | G200.00 |
| Benign essential hypertension | G201.00 |
| Systolic hypertension | G202.00 |
| Diastolic hypertension | G203.00 |
| Essential hypertension NOS | G20z.00 |
| Hypertension NOS | G20z.11 |
| Hypertensive heart disease | G21..00 |
| Malignant hypertensive heart disease | G210.00 |
| Malignant hypertensive heart disease without CCF | G210000 |
| Malignant hypertensive heart disease with CCF | G210100 |
| Benign hypertensive heart disease | G211.00 |
| Benign hypertensive heart disease without CCF | G211000 |
| Benign hypertensive heart disease with CCF | G211100 |
| Hypertensive heart disease NOS | G21z.00 |
| Hypertensive heart disease NOS without CCF | G21z000 |
| Cardiomegaly - hypertensive | G21z011 |
| Hypertensive heart disease NOS with CCF | G21z100 |
| Hypertensive heart disease NOS | G21zz00 |
| Hypertensive renal disease | G22..00 |
| Malignant hypertensive renal disease | G220.00 |
| Benign hypertensive renal disease | G221.00 |
| Hypertensive renal disease with renal failure | G222.00 |
| Hypertensive renal disease NOS | G22z.00 |
| Renal hypertension | G22z.11 |
| Hypertensive heart and renal disease | G23..00 |
| Malignant hypertensive heart and renal disease | G230.00 |
| Benign hypertensive heart and renal disease | G231.00 |
| Hypertensive heart&renal dis wth (congestive) heart failure | G232.00 |
| Hypertensive heart and renal disease with renal failure | G233.00 |
| Hyperten heart&renal dis+both(congestv)heart and renal fail | G234.00 |
| Hypertensive heart and renal disease NOS | G23z.00 |
| Other specified hypertensive disease | G2y..00 |
| Hypertensive disease NOS | G2z..00 |
| Hypertensive encephalopathy | G672.00 |
| Hypertensive crisis | G672.11 |
| [X]Hypertensive diseases | Gyu2.00 |
| Other pre-existing hypertension in preg/childbirth/puerp | L122.00 |
| Other pre-existing hypertension in preg/childb/puerp unspec | L122000 |
| Other pre-existing hypertension in preg/childb/puerp - deliv | L122100 |
| Other pre-exist hypertension in preg/childb/puerp-not deliv | L122300 |
| Other pre-existing hypertension in preg/childb/puerp NOS | L122z00 |
| Pre-eclampsia or eclampsia with pre-existing hypertension | L127.00 |
| Pre-eclampsia or eclampsia + pre-existing hypertension NOS | L127z00 |
| Pre-exist hypertension compl preg childbirth and puerperium | L128.00 |
| Pre-exist hyperten heart dis compl preg childbth+puerperium | L128000 |
| Pre-exist 2ndry hypertens comp preg childbth and puerperium | L128200 |
| Adverse reaction to other antihypertensives | TJC7.00 |
| Adverse reaction to antihypertensives NOS | TJC7z00 |
| [X]Oth antihyperten drug caus advers eff in therap use, NEC | U60C500 |
| [X] Adverse reaction to other antihypertensives | U60C511 |
| [X] Adverse reaction to antihypertensives NOS | U60C51A |
| Hypertension induced by oral contraceptive pill | 6146200 |
| Secondary hypertension | G24..00 |
| Secondary malignant hypertension | G240.00 |
| Secondary malignant renovascular hypertension | G240000 |
| Secondary malignant hypertension NOS | G240z00 |
| Secondary benign hypertension | G241.00 |
| Secondary benign renovascular hypertension | G241000 |
| Secondary benign hypertension NOS | G241z00 |
| Hypertension secondary to endocrine disorders | G244.00 |
| Secondary hypertension NOS | G24z.00 |
| Secondary renovascular hypertension NOS | G24z000 |
| Hypertension secondary to drug | G24z100 |
| Secondary hypertension NOS | G24zz00 |
| [X]Hypertension secondary to other renal disorders | Gyu2100 |

Text S3. Definition of the smoking status of patients.

| A patient was classified as a “current/ex- smoker” if there was a record of the following codes up to the index date ICD-10 codes (F17, Z716, Z720) or record of prescription related to cessation of smoking (BNF chapter 4.10.2) or manual entry of record of smoking status or read codes in **Supplementary table S3**. If none were found then the patient was classified as “never smoked”. Phenotypes from CALIBER can be accessed at <https://phenotypes.healthdatagateway.org/phenotypes/?collection_ids=21> |
| --- |

Table S3. Read codes to identify patients that are current or ex- smokers.

| Read Term | Read Code |
| --- | --- |
| Ex-trivial smoker (<1/day) | 1377 |
| Ex-light smoker (1-9/day) | 1378 |
| Ex-moderate smoker (10-19/day) | 1379 |
| Ex-heavy smoker (20-39/day) | 137A.00 |
| Ex-very heavy smoker (40+/day) | 137B.00 |
| Ex-smoker - amount unknown | 137F.00 |
| Stopped smoking | 137K.00 |
| Ex pipe smoker | 137N.00 |
| Ex cigar smoker | 137O.00 |
| Ex smoker | 137S.00 |
| Date ceased smoking | 137T.00 |
| Ex-cigarette smoker | 137j.00 |
| Ex-smoker annual review - enhanced services administration | 9km..00 |
| Tobacco dependence in remission | E251300 |
| Cigarette pack-years | 137g.00 |
| Pack years | 388B.00 |
| Smokers' cough | H310100 |
| Pack years | ZRb3.00 |
| [V]Personal history of tobacco abuse | ZV11600 |
| Tobacco consumption | 137..00 |
| Smoker - amount smoked | 137..11 |
| Trivial smoker - < 1 cig/day | 1372 |
| Occasional smoker | 1372.11 |
| Light smoker - 1-9 cigs/day | 1373 |
| Moderate smoker - 10-19 cigs/d | 1374 |
| Heavy smoker - 20-39 cigs/day | 1375 |
| Very heavy smoker - 40+cigs/d | 1376 |
| Keeps trying to stop smoking | 137C.00 |
| Trying to give up smoking | 137G.00 |
| Pipe smoker | 137H.00 |
| Cigar smoker | 137J.00 |
| Rolls own cigarettes | 137M.00 |
| Cigarette smoker | 137P.00 |
| Smoker | 137P.11 |
| Smoking started | 137Q.00 |
| Smoking restarted | 137Q.11 |
| Current smoker | 137R.00 |
| Smoking reduced | 137V.00 |
| Cigarette consumption | 137X.00 |
| Cigar consumption | 137Y.00 |
| Tobacco consumption NOS | 137Z.00 |
| Pipe tobacco consumption | 137a.00 |
| Ready to stop smoking | 137b.00 |
| Thinking about stopping smoking | 137c.00 |
| Not interested in stopping smoking | 137d.00 |
| Smoking restarted | 137e.00 |
| Reason for restarting smoking | 137f.00 |
| Minutes from waking to first tobacco consumption | 137h.00 |
| Smoking cessation milestones | 13p..00 |
| Negotiated date for cessation of smoking | 13p0.00 |
| Smoking status at 4 weeks | 13p1.00 |
| Smoking status between 4 and 52 weeks | 13p2.00 |
| Smoking status at 52 weeks | 13p3.00 |
| Smoking free weeks | 13p4.00 |
| Smoking cessation programme start date | 13p5.00 |
| Carbon monoxide reading at 4 weeks | 13p6.00 |
| Fagerstrom test for nicotine dependence | 38DH.00 |
| Health ed. - smoking | 6791 |
| Pregnancy smoking advice | 67A3.00 |
| Lifestyle advice regarding smoking | 67H1.00 |
| Brief intervention for smoking cessation | 67H6.00 |
| Smoking cessation therapy | 745H.00 |
| Nicotine replacement therapy using nicotine patches | 745H000 |
| Nicotine replacement therapy using nicotine gum | 745H100 |
| Nicotine replacement therapy using nicotine inhalator | 745H200 |
| Nicotine replacement therapy using nicotine lozenges | 745H300 |
| Smoking cessation drug therapy | 745H400 |
| Other specified smoking cessation therapy | 745Hy00 |
| Smoking cessation therapy NOS | 745Hz00 |
| Nicotine replacement therapy | 8B2B.00 |
| Over the counter nicotine replacement therapy | 8B3Y.00 |
| Nicotine replacement therapy provided free | 8B3f.00 |
| Nicotine replacement therapy provided by community pharmacis | 8BP3.00 |
| Smoking cessation advice | 8CAL.00 |
| Smoking cessation advice provided by community pharmacist | 8CAg.00 |
| Referral to smoking cessation advisor | 8H7i.00 |
| Stop smoking face to face follow-up | 8HBM.00 |
| Referral to stop-smoking clinic | 8HTK.00 |
| Referral to NHS stop smoking service | 8HkQ.00 |
| Nicotine replacement therapy contraindicated | 8I2I.00 |
| Bupropion contraindicated | 8I2J.00 |
| Nicotine replacement therapy refused | 8I39.00 |
| Bupropion refused | 8I3M.00 |
| Seen by smoking cessation advisor | 9N2k.00 |
| DNA - Did not attend smoking cessation clinic | 9N4M.00 |
| Anti-smoking monitoring admin. | 9OO..00 |
| Stop smoking clinic admin. | 9OO..11 |
| Stop smoking monitoring admin. | 9OO..12 |
| Attends stop smoking monitor. | 9OO1.00 |
| Refuses stop smoking monitor | 9OO2.00 |
| Stop smoking monitor default | 9OO3.00 |
| Stop smoking monitor 1st lettr | 9OO4.00 |
| Stop smoking monitor 2nd lettr | 9OO5.00 |
| Stop smoking monitor 3rd lettr | 9OO6.00 |
| Stop smoking monitor verb.inv. | 9OO7.00 |
| Stop smoking monitor phone inv | 9OO8.00 |
| Stop smoking monitoring delete | 9OO9.00 |
| Stop smoking monitor.chck done | 9OOA.00 |
| Stop smoking monitor admin.NOS | 9OOZ.00 |
| Smoking cessation - enhanced services administration | 9kc..00 |
| Smoking cessatn monitor template complet - enhanc serv admin | 9kc0.00 |
| Current smoker annual review - enhanced services admin | 9ko..00 |
| Nicotine withdrawal | E023.00 |
| Tobacco dependence | E251.00 |
| Tobacco dependence, unspecified | E251000 |
| Tobacco dependence, continuous | E251100 |
| Tobacco dependence NOS | E251z00 |
| [X]Mental and behavioural disorder due to use of tobacco | Eu17.00 |
| [X]Mental and behav dis due to use of tobacco: harmful use | Eu17100 |
| Advice on smoking | ZG23300 |
| Fagerstrom test for nicotine dependence | ZRBm200 |
| FTND - Fagerstrom test for nicotine dependence | ZRBm211 |
| Motives for smoking scale | ZRaM.00 |
| Occasions for smoking scale | ZRao.00 |
| Reasons for smoking scale | ZRh4.00 |
| RFS - Reasons for smoking scale | ZRh4.11 |
| [V]Tobacco use | ZV4K000 |
| [V]Tobacco abuse counselling | ZV6D800 |

Text S4. Definition of the alcohol drinking status of patients.

| A patient was classified in their relevant category (non-drinker, ex-drinker, current-drinker) if there was a record of the following codes up to the index date read codes in **Supplementary table S4** or manual entry of record of alcohol drinking status. If none were found then the patient had a “missing” drinking status. Phenotypes from CALIBER can be accessed at <https://phenotypes.healthdatagateway.org/phenotypes/?collection_ids=21> |
| --- |

Table S4. Read codes to identify alcohol drinking status of patients.

| Category | Read Term | Read Code |
| --- | --- | --- |
| None | Teetotaller | 1361 |
| None | Non drinker alcohol | 1361.11 |
| None | Non-drinker alcohol | 1361.12 |
| None | Current non drinker | 136M.00 |
| Ex | Stopped drinking alcohol | 1367 |
| Ex | Ex-trivial drinker (<1u/day) | 136A.00 |
| Ex | Ex-light drinker - (1-2u/day) | 136B.00 |
| Ex | Ex-moderate drinker - (3-6u/d) | 136C.00 |
| Ex | Ex-heavy drinker - (7-9u/day) | 136D.00 |
| Ex | Ex-very heavy drinker-(>9u/d) | 136E.00 |
| Current | Drinks rarely | 1362.11 |
| Current | Drinks occasionally | 1362.12 |
| Current | Spirit drinker | 136F.00 |
| Current | Beer drinker | 136G.00 |
| Current | Drinks beer and spirits | 136H.00 |
| Current | Drinks wine | 136I.00 |
| Current | Social drinker | 136J.00 |
| Current | Alcohol intake within recommended sensible limits | 136L.00 |
| Current | Light drinker | 136N.00 |
| Current | Moderate drinker | 136O.00 |
| Current | Pain in lymph nodes after alcohol consumption | 1D19.00 |
| Current | O/E - breath - alcohol smell | 2577 |
| Current | O/E - alcoholic breath | 2577.11 |
| Current | Drunkenness NOS | E250.11 |
| Current | Hangover (alcohol) | E250.12 |
| Current | Inebriety NOS | E250.13 |
| Current | Intoxication - alcohol | E250.14 |
| Current | [D]Alcohol blood level excessive | R103.00 |
| Current | [X]Evid of alcohol involv determind by level of intoxication | U81..00 |
| Current | Alcohol intake above recommended sensible limits | 136K.00 |
| Current | Heavy drinker | 136P.00 |
| Current | Very heavy drinker | 136Q.00 |
| Current | Hazardous alcohol use | 136S.00 |
| Current | Harmful alcohol use | 136T.00 |
| Current | Alcohol misuse | 136W.00 |
| Current | Disqualified from driving due to excess alcohol | 13ZY.00 |
| Current | Alcohol problem drinking | E23..12 |
| Current | Nondependent alcohol abuse | E250.00 |
| Current | Nondependent alcohol abuse, unspecified | E250000 |
| Current | Nondependent alcohol abuse, continuous | E250100 |
| Current | Nondependent alcohol abuse in remission | E250300 |
| Current | Nondependent alcohol abuse NOS | E250z00 |
| Current | [V]Problems related to lifestyle alcohol use | ZV11311 |
| Current | Binge drinker | 136R.00 |
| Current | Nondependent alcohol abuse, episodic | E250200 |
| Current | Trivial drinker - <1u/day | 1362 |
| Current | Light drinker - 1-2u/day | 1363 |
| Current | Moderate drinker - 3-6u/day | 1364 |
| Current | Heavy drinker - 7-9u/day | 1365 |
| Current | Very heavy drinker - >9u/day | 1366 |

Table S5. ICD-10 codes of cancer outcomes and categorisation into anatomical regions and organ systems.

| ICD-10 (3 digits) | ICD-10 (4 digits) | Body region | Organ system |
| --- | --- | --- | --- |
| C00 | C000 | Head and neck | Other |
|  | C001 | Head and neck | Other |
|  | C002 | Head and neck | Other |
|  | C003 | Head and neck | Other |
|  | C004 | Head and neck | Other |
|  | C006 | Head and neck | Other |
|  | C009 | Head and neck | Other |
| C01 | C01 | Head and neck | Other |
| C02 | C020 | Head and neck | Other |
|  | C021 | Head and neck | Other |
|  | C022 | Head and neck | Other |
|  | C023 | Head and neck | Other |
|  | C024 | Head and neck | Other |
|  | C028 | Head and neck | Other |
|  | C029 | Head and neck | Other |
| C03 | C030 | Head and neck | Other |
|  | C031 | Head and neck | Other |
|  | C039 | Head and neck | Other |
| C04 | C040 | Head and neck | Other |
|  | C041 | Head and neck | Other |
|  | C049 | Head and neck | Other |
| C05 | C050 | Head and neck | Other |
|  | C051 | Head and neck | Other |
|  | C052 | Head and neck | Other |
|  | C058 | Head and neck | Other |
|  | C059 | Head and neck | Other |
| C06 | C060 | Head and neck | Other |
|  | C061 | Head and neck | Other |
|  | C062 | Head and neck | Other |
|  | C069 | Head and neck | Other |
| C07 | C07 | Head and neck | Other |
| C08 | C080 | Head and neck | Other |
|  | C089 | Head and neck | Other |
| C09 | C090 | Head and neck | Other |
|  | C098 | Head and neck | Other |
|  | C099 | Head and neck | Other |
| C10 | C100 | Head and neck | Other |
|  | C101 | Head and neck | Other |
|  | C103 | Head and neck | Other |
| C10 | C109 | Head and neck | Other |
| C11 | C111 | Head and neck | Respiratory |
|  | C112 | Head and neck | Respiratory |
|  | C119 | Head and neck | Digestive |
| C12 | C12 | Head and neck | Other |
| C13 | C130 | Head and neck | Other |
|  | C131 | Head and neck | Other |
|  | C132 | Head and neck | Other |
|  | C139 | Head and neck | Other |
| C14 | C140 | Head and neck | Other |
|  | C148 | Head and neck | Other |
| C15 | C150 | Chest | Digestive |
|  | C151 | Chest | Digestive |
|  | C153 | Chest | Digestive |
|  | C154 | Chest | Digestive |
|  | C155 | Chest | Digestive |
|  | C158 | Chest | Digestive |
|  | C159 | Chest | Digestive |
| C16 | C160 | Abdomen | Digestive |
|  | C161 | Abdomen | Digestive |
|  | C162 | Abdomen | Digestive |
|  | C163 | Abdomen | Digestive |
|  | C164 | Abdomen | Digestive |
|  | C165 | Abdomen | Digestive |
|  | C166 | Abdomen | Digestive |
|  | C168 | Abdomen | Digestive |
|  | C169 | Abdomen | Digestive |
| C17 | C170 | Abdomen | Digestive |
|  | C171 | Abdomen | Digestive |
|  | C172 | Abdomen | Digestive |
|  | C179 | Abdomen | Digestive |
| C18 | C180 | Abdomen | Digestive |
|  | C181 | Abdomen | Digestive |
|  | C182 | Abdomen | Digestive |
|  | C183 | Abdomen | Digestive |
|  | C184 | Abdomen | Digestive |
|  | C185 | Abdomen | Digestive |
|  | C186 | Abdomen | Digestive |
|  | C187 | Pelvis | Digestive |
|  | C188 | Abdomen | Digestive |
|  | C189 | Abdomen | Digestive |
| C19 | C19 | Pelvis | Digestive |
| C20 | C20 | Pelvis | Digestive |
| C21 | C210 | Pelvis | Digestive |
|  | C211 | Pelvis | Digestive |
|  | C218 | Pelvis | Digestive |
| C22 | C220 | Abdomen | Digestive |
|  | C221 | Abdomen | Digestive |
| C22 | C222 | Abdomen | Digestive |
|  | C223 | Abdomen | Digestive |
|  | C224 | Abdomen | Digestive |
|  | C227 | Abdomen | Digestive |
|  | C229 | Abdomen | Digestive |
| C23 | C23 | Abdomen | Digestive |
| C24 | C240 | Abdomen | Digestive |
|  | C241 | Abdomen | Digestive |
|  | C249 | Abdomen | Digestive |
| C25 | C250 | Abdomen | Digestive |
|  | C251 | Abdomen | Digestive |
|  | C252 | Abdomen | Digestive |
|  | C253 | Abdomen | Digestive |
|  | C257 | Abdomen | Digestive |
|  | C258 | Abdomen | Digestive |
|  | C259 | Abdomen | Digestive |
| C26 | C260 | Abdomen | Digestive |
|  | C269 | Abdomen | Other |
| C30 | C300 | Head and neck | Other |
|  | C301 | Head and neck | Other |
| C31 | C310 | Head and neck | Other |
|  | C311 | Head and neck | Other |
|  | C313 | Head and neck | Other |
| C32 | C320 | Head and neck | Respiratory |
|  | C321 | Head and neck | Respiratory |
|  | C322 | Head and neck | Respiratory |
|  | C323 | Head and neck | Respiratory |
|  | C329 | Head and neck | Respiratory |
| C33 | C33 | Chest | Respiratory |
| C34 | C340 | Chest | Respiratory |
|  | C341 | Chest | Respiratory |
|  | C342 | Chest | Respiratory |
|  | C343 | Chest | Respiratory |
|  | C348 | Chest | Respiratory |
|  | C349 | Chest | Respiratory |
| C37 | C37 | Chest | Immune & haematological |
| C38 | C380 | Chest | Cardiovascular |
|  | C381 | Chest | Cardiovascular |
|  | C383 | Chest | Respiratory |
|  | C384 | Chest | Respiratory |
|  | C388 | Chest | Other |
| C39 | C399 | Chest | Respiratory |
| C40 | C400 | Upper and lower limbs | Skeletal |
|  | C402 | Upper and lower limbs | Skeletal |
| C40 | C403 | Upper and lower limbs | Skeletal |
|  | C409 | Upper and lower limbs | Skeletal |
| C41 | C410 | Head and neck | Skeletal |
|  | C412 | Other | Skeletal |
|  | C413 | Chest | Skeletal |
|  | C414 | Pelvis | Skeletal |
|  | C418 | Other | Skeletal |
|  | C419 | Other | Skeletal |
| C43 | C430 | Head and neck | Other |
|  | C431 | Head and neck | Other |
|  | C432 | Head and neck | Other |
|  | C433 | Head and neck | Other |
|  | C434 | Head and neck | Other |
|  | C435 | Other | Other |
|  | C436 | Upper and lower limbs | Other |
|  | C437 | Upper and lower limbs | Other |
|  | C438 | Other | Other |
|  | C439 | Other | Other |
| C45 | C450 | Chest | Respiratory |
|  | C451 | Abdomen | Digestive |
|  | C452 | Chest | Cardiovascular |
|  | C457 | Other | Other |
|  | C459 | Other | Other |
| C46 | C461 | Other | Other |
| C47 | C470 | Head and neck | Other |
|  | C472 | Upper and lower limbs | Other |
|  | C479 | Other | Other |
| C48 | C480 | Abdomen | Other |
|  | C481 | Abdomen | Other |
|  | C482 | Abdomen | Other |
|  | C488 | Abdomen | Other |
| C49 | C490 | Head and neck | Other |
|  | C491 | Upper and lower limbs | Other |
|  | C492 | Upper and lower limbs | Other |
|  | C493 | Chest | Other |
|  | C494 | Abdomen | Other |
|  | C495 | Pelvis | Other |
|  | C496 | Pelvis | Other |
|  | C499 | Other | Other |
| C50 | C500 | Chest | Breast |
|  | C501 | Chest | Breast |
|  | C502 | Chest | Breast |
| C50 | C503 | Chest | Breast |
|  | C504 | Chest | Breast |
|  | C505 | Chest | Breast |
|  | C506 | Chest | Breast |
|  | C508 | Chest | Breast |
|  | C509 | Chest | Breast |
| C51 | C510 | Pelvis | Reproductive |
|  | C511 | Pelvis | Reproductive |
|  | C512 | Pelvis | Reproductive |
|  | C518 | Pelvis | Reproductive |
|  | C519 | Pelvis | Reproductive |
| C52 | C52 | Pelvis | Reproductive |
| C53 | C530 | Pelvis | Reproductive |
|  | C531 | Pelvis | Reproductive |
|  | C539 | Pelvis | Reproductive |
| C54 | C540 | Pelvis | Reproductive |
|  | C541 | Pelvis | Reproductive |
|  | C542 | Pelvis | Reproductive |
|  | C549 | Pelvis | Reproductive |
| C55 | C55 | Pelvis | Reproductive |
| C56 | C56 | Pelvis | Reproductive |
| C57 | C570 | Pelvis | Reproductive |
|  | C578 | Pelvis | Reproductive |
|  | C579 | Pelvis | Reproductive |
| C60 | C600 | Pelvis | Reproductive |
|  | C601 | Pelvis | Reproductive |
|  | C602 | Pelvis | Reproductive |
|  | C608 | Pelvis | Reproductive |
|  | C609 | Pelvis | Reproductive |
| C61 | C61 | Pelvis | Reproductive |
| C62 | C621 | Pelvis | Reproductive |
| C62 | C629 | Pelvis | Reproductive |
| C63 | C631 | Pelvis | Reproductive |
|  | C632 | Pelvis | Urinary |
|  | C637 | Pelvis | Reproductive |
| C64 | C64 | Abdomen | Urinary |
| C65 | C65 | Abdomen | Urinary |
| C66 | C66 | Abdomen | Urinary |
| C67 | C670 | Pelvis | Urinary |
|  | C671 | Pelvis | Urinary |
|  | C672 | Pelvis | Urinary |
|  | C673 | Pelvis | Urinary |
|  | C674 | Pelvis | Urinary |
|  | C675 | Pelvis | Urinary |
| C67 | C676 | Pelvis | Urinary |
|  | C678 | Pelvis | Urinary |
|  | C679 | Pelvis | Urinary |
| C68 | C680 | Pelvis | Urinary |
|  | C688 | Pelvis | Urinary |
|  | C689 | Pelvis | Urinary |
| C69 | C690 | Head and neck | Other |
|  | C692 | Head and neck | Other |
|  | C693 | Head and neck | Other |
|  | C694 | Head and neck | Other |
|  | C695 | Head and neck | Other |
|  | C698 | Head and neck | Other |
|  | C699 | Head and neck | Other |
| C70 | C700 | Head and neck | Nervous |
|  | C701 | Brain/CNS | Nervous |
|  | C709 | Brain/CNS | Nervous |
| C71 | C710 | Brain/CNS | Nervous |
|  | C711 | Brain/CNS | Nervous |
|  | C712 | Brain/CNS | Nervous |
|  | C713 | Brain/CNS | Nervous |
|  | C714 | Brain/CNS | Nervous |
|  | C715 | Brain/CNS | Nervous |
|  | C716 | Brain/CNS | Nervous |
|  | C717 | Brain/CNS | Nervous |
|  | C718 | Brain/CNS | Nervous |
|  | C719 | Brain/CNS | Nervous |
| C72 | C720 | Brain/CNS | Nervous |
|  | C725 | Head and neck | Nervous |
| C73 | C73 | Head and neck | Endocrine |
| C74 | C740 | Abdomen | Endocrine |
|  | C741 | Abdomen | Endocrine |
|  | C749 | Abdomen | Endocrine |
| C75 | C750 | Head and neck | Endocrine |
|  | C751 | Brain/CNS | Endocrine |
| C76 | C760 | Head and neck | Other |
|  | C761 | Chest | Other |
|  | C762 | Abdomen | Other |
|  | C763 | Pelvis | Other |
|  | C764 | Upper and lower limbs | Other |
|  | C765 | Upper and lower limbs | Other |
|  | C767 | Other | Other |
| C77 | C770 | Unknown primary | Unknown primary |
|  | C771 | Unknown primary | Unknown primary |
| C77 | C772 | Unknown primary | Unknown primary |
|  | C773 | Unknown primary | Unknown primary |
|  | C774 | Unknown primary | Unknown primary |
|  | C778 | Unknown primary | Unknown primary |
|  | C779 | Unknown primary | Unknown primary |
| C78 | C780 | Unknown primary | Unknown primary |
|  | C781 | Unknown primary | Unknown primary |
|  | C782 | Unknown primary | Unknown primary |
|  | C784 | Unknown primary | Unknown primary |
|  | C785 | Unknown primary | Unknown primary |
|  | C786 | Unknown primary | Unknown primary |
|  | C787 | Unknown primary | Unknown primary |
|  | C788 | Unknown primary | Unknown primary |
| C79 | C790 | Unknown primary | Unknown primary |
|  | C791 | Unknown primary | Unknown primary |
|  | C792 | Unknown primary | Unknown primary |
|  | C793 | Unknown primary | Unknown primary |
|  | C794 | Unknown primary | Unknown primary |
|  | C795 | Unknown primary | Unknown primary |
|  | C796 | Unknown primary | Unknown primary |
|  | C797 | Unknown primary | Unknown primary |
|  | C798 | Unknown primary | Unknown primary |
| C80 | C80 | Unknown primary | Unknown primary |
| C81 | C810 | Other | Immune & haematological |
|  | C811 | Other | Immune & haematological |
|  | C812 | Other | Immune & haematological |
|  | C819 | Other | Immune & haematological |
| C82 | C820 | Other | Immune & haematological |
|  | C821 | Other | Immune & haematological |
|  | C822 | Other | Immune & haematological |
|  | C827 | Other | Immune & haematological |
|  | C829 | Other | Immune & haematological |
| C83 | C830 | Other | Immune & haematological |
|  | C831 | Other | Immune & haematological |
|  | C833 | Other | Immune & haematological |
| C83 | C834 | Other | Immune & haematological |
|  | C835 | Other | Immune & haematological |
|  | C837 | Other | Immune & haematological |
|  | C838 | Other | Immune & haematological |
|  | C839 | Other | Immune & haematological |
| C84 | C840 | Other | Immune & haematological |
|  | C841 | Other | Immune & haematological |
|  | C844 | Other | Immune & haematological |
|  | C845 | Other | Immune & haematological |
| C85 | C851 | Other | Immune & haematological |
|  | C857 | Other | Immune & haematological |
|  | C859 | Other | Immune & haematological |
| C88 | C880 | Other | Immune & haematological |
|  | C889 | Other | Immune & haematological |
| C90 | C900 | Other | Immune & haematological |
|  | C901 | Other | Immune & haematological |
|  | C902 | Other | Immune & haematological |
| C91 | C910 | Other | Immune & haematological |
|  | C911 | Other | Immune & haematological |
|  | C913 | Other | Immune & haematological |
|  | C914 | Other | Immune & haematological |
|  | C915 | Other | Immune & haematological |
|  | C919 | Other | Immune & haematological |
| C92 | C920 | Other | Immune & haematological |
|  | C921 | Other | Immune & haematological |
|  | C923 | Other | Immune & haematological |
|  | C924 | Other | Immune & haematological |
|  | C925 | Other | Immune & haematological |
|  | C927 | Other | Immune & haematological |
|  | C929 | Other | Immune & haematological |
| C93 | C930 | Other | Immune & haematological |
|  | C931 | Other | Immune & haematological |
| C94 | C940 | Other | Immune & haematological |
|  | C942 | Other | Immune & haematological |
| C94 | C944 | Other | Immune & haematological |
| C95 | C950 | Other | Immune & haematological |
|  | C951 | Other | Immune & haematological |
|  | C957 | Other | Immune & haematological |
|  | C959 | Other | Immune & haematological |
| C96 | C960 | Other | Immune & haematological |
|  | C961 | Other | Immune & haematological |
|  | C969 | Other | Immune & haematological |
| C97 | C97 | Other | Other |


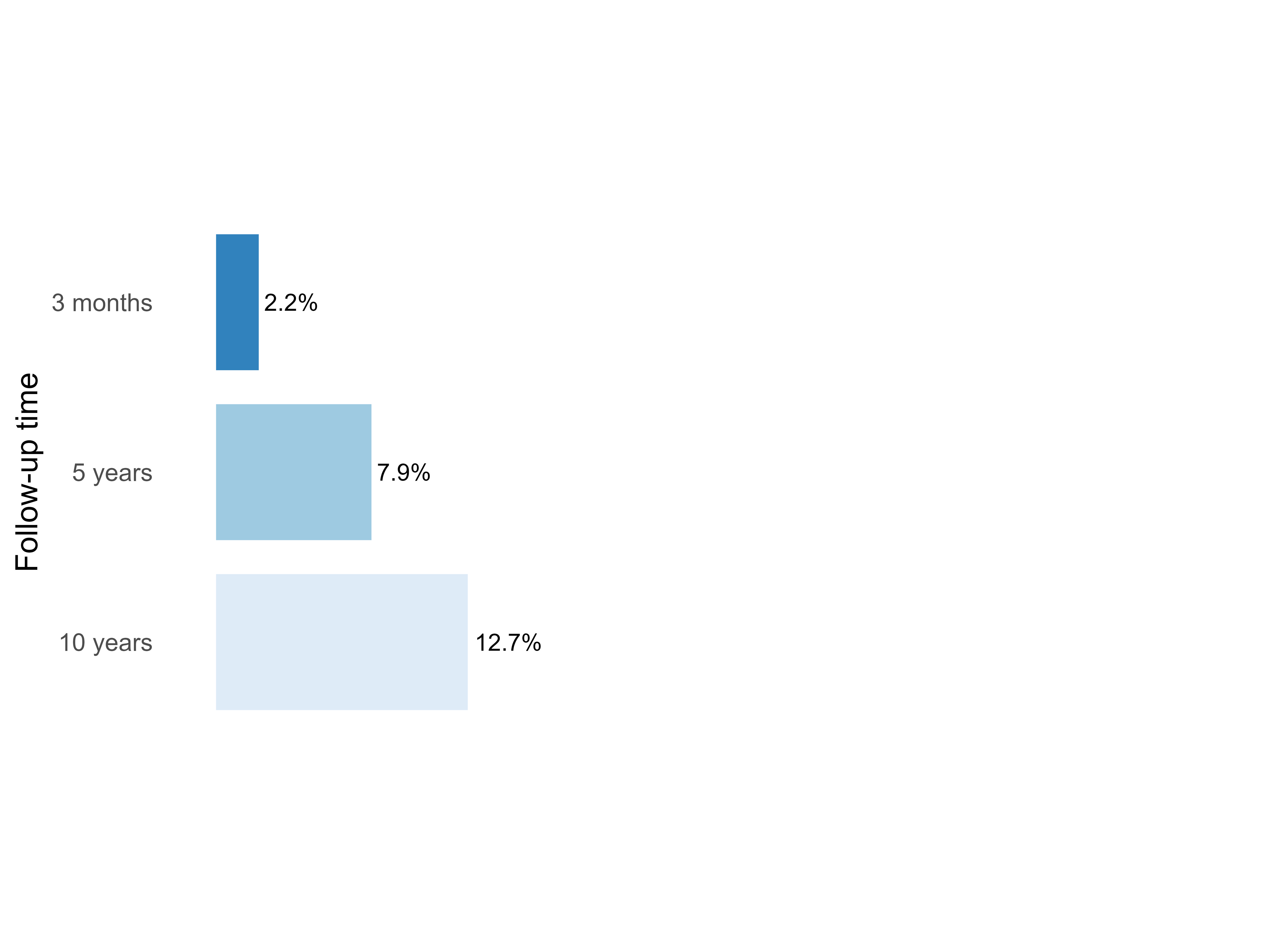


Figure S1. Incidence of cancer after new-onset atrial fibrillation. Percentages represent the proportion of cancer incidence at 3 months, and the cumulative incidence of cancer at 5 years and 10 years. 2.2% of patients with new-onset atrial fibrillation were diagnosed with cancer within 3 months. After accounting for censoring, cumulative incidence of cancer in patients with new-onset AF was 7.9% at 5 years and 12.7% at 10 years.

Table S6. Proportion of outcomes at different time points for patients with new-onset atrial fibrillation (AF) and their matched controls, split by men and women.

|  | | | **Men  n (%*)** | | **Women n (%*)** | |
| --- | --- | --- | --- | --- | --- | --- |
| **Follow-up time** |  |  | **Control** | **AF** | **Control** | **AF** |
| <= 3 months |  |  |  |  |  |  |
|  | Body Region | Abdomen | 62 (0.10%) | 324 (0.55%) | 46 (0.08%) | 244 (0.42%) |
|  |  | Brain/CNS | n<5 (0.01%) | 23 (0.04%) | n<5 (0.01%) | 14 (0.02%) |
|  |  | Chest | 56 (0.09%) | 465 (0.78%) | 106 (0.18%) | 416 (0.72%) |
|  |  | Head and neck | 9 (0.02%) | 37 (0.06%) | 5 (0.01%) | 17 (0.03%) |
|  |  | Other | 25 (0.04%) | 146 (0.25%) | 22 (0.04%) | 125 (0.22%) |
|  |  | Pelvis | 127 (0.21%) | 348 (0.59%) | 51 (0.09%) | 206 (0.36%) |
|  |  | Unknown primary | 11 (0.02%) | 83 (0.14%) | 14 (0.02%) | 87 (0.15%) |
|  |  | Upper and lower limbs | n<5 (0.01%) | 6 (0.01%) | 5 (0.01%) | n<5 (0.01%) |
|  | Organ System | Breast | n<5 (0.01%) | n<5 (0.01%) | 61 (0.11%) | 110 (0.19%) |
|  |  | Cardiovascular | n<5 (0.01%) | n<5 (0.01%) | n<5 (0.01%) | n<5 (0.01%) |
|  |  | Digestive | 86 (0.15%) | 378 (0.64%) | 60 (0.10%) | 320 (0.55%) |
|  |  | Endocrine | n<5 (0.01%) | 5 (0.01%) | n<5 (0.01%) | 7 (0.01%) |
|  |  | Immune & haematological | 23 (0.04%) | 142 (0.24%) | 22 (0.04%) | 117 (0.20%) |
|  |  | Nervous | n<5 (0.01%) | 23 (0.04%) | n<5 (0.01%) | 15 (0.03%) |
|  |  | Other | 14 (0.02%) | 38 (0.06%) | 15 (0.03%) | 33 (0.06%) |
|  |  | Reproductive | 79 (0.13%) | 175 (0.30%) | 26 (0.04%) | 81 (0.14%) |
|  |  | Respiratory | 47 (0.08%) | 439 (0.74%) | 37 (0.06%) | 278 (0.48%) |
|  |  | Skeletal | n<5 (0.01%) | n<5 (0.01%) | n<5 (0.01%) | n<5 (0.01%) |
|  |  | Unknown primary | 11 (0.02%) | 83 (0.14%) | 14 (0.02%) | 87 (0.15%) |
|  |  | Urinary | 32 (0.05%) | 146 (0.25%) | 11 (0.02%) | 60 (0.10%) |
|  |  |  |  |  |  |  |
| 3 months - 5 years |  |  |  |  |  |  |
|  | Body Region | Abdomen | 775 (1.33%) | 877 (1.67%) | 663 (1.17%) | 657 (1.34%) |
|  |  | Brain/CNS | 53 (0.09%) | 54 (0.10%) | 30 (0.05%) | 26 (0.05%) |
|  |  | Chest | 891 (1.53%) | 913 (1.74%) | 1212 (2.14%) | 1170 (2.39%) |
|  |  | Head and neck | 162 (0.28%) | 184 (0.35%) | 90 (0.16%) | 94 (0.19%) |
|  |  | Other | 423 (0.72%) | 438 (0.83%) | 264 (0.47%) | 261 (0.53%) |
|  |  | Pelvis | 1904 (3.27%) | 1628 (3.10%) | 602 (1.06%) | 590 (1.20%) |
|  |  | Unknown primary | 177 (0.30%) | 193 (0.37%) | 202 (0.36%) | 206 (0.42%) |
|  |  | Upper and lower limbs | 43 (0.07%) | 42 (0.08%) | 64 (0.11%) | 46 (0.09%) |
|  | Organ System | Breast | 11 (0.02%) | 8 (0.02%) | 701 (1.24%) | 659 (1.34%) |
|  |  | Cardiovascular | n<5 (0.01%) | n<5 (0.01%) | n<5 (0.01%) | n<5 (0.01%) |
|  |  | Digestive | 1183 (2.03%) | 1189 (2.26%) | 889 (1.57%) | 861 (1.76%) |
|  |  | Endocrine | 10 (0.02%) | 13 (0.02%) | 13 (0.02%) | 19 (0.04%) |
|  |  | Immune & haematological | 369 (0.63%) | 381 (0.72%) | 253 (0.45%) | 245 (0.50%) |
|  |  | Nervous | 53 (0.09%) | 55 (0.10%) | 31 (0.05%) | 27 (0.06%) |
|  |  | Other | 227 (0.39%) | 241 (0.46%) | 164 (0.29%) | 151 (0.31%) |
|  |  | Reproductive | 1261 (2.16%) | 1087 (2.06%) | 281 (0.50%) | 279 (0.57%) |
|  |  | Respiratory | 756 (1.29%) | 789 (1.50%) | 426 (0.75%) | 436 (0.89%) |
|  |  | Skeletal | n<5 (0.01%) | n<5 (0.01%) | n<5 (0.01%) | n<5 (0.01%) |
|  |  | Unknown primary | 177 (0.30%) | 193 (0.37%) | 202 (0.36%) | 206 (0.42%) |
|  |  | Urinary | 395 (0.68%) | 383 (0.73%) | 165 (0.29%) | 166 (0.34%) |
|  |  |  |  |  |  |  |
| > 5 years |  |  |  |  |  |  |
|  | Body Region | Abdomen | 498 (1.58%) | 509 (2.02%) | 372 (1.31%) | 304 (1.44%) |
|  |  | Brain/CNS | 32 (0.10%) | 25 (0.10%) | 20 (0.07%) | 8 (0.04%) |
|  |  | Chest | 551 (1.74%) | 495 (1.96%) | 721 (2.56%) | 574 (2.76%) |
|  |  | Head and neck | 115 (0.36%) | 104 (0.41%) | 68 (0.24%) | 36 (0.17%) |
|  |  | Other | 263 (0.83%) | 254 (1.01%) | 171 (0.60%) | 119 (0.56%) |
|  |  | Pelvis | 1170 (3.81%) | 947 (3.86%) | 326 (1.15%) | 263 (1.25%) |
|  |  | Unknown primary | 103 (0.32%) | 89 (0.35%) | 92 (0.32%) | 83 (0.39%) |
|  |  | Upper and lower limbs | 25 (0.08%) | 23 (0.09%) | 40 (0.14%) | 24 (0.11%) |
|  | Organ System | Breast | 5 (0.02%) | 6 (0.02%) | 424 (1.50%) | 356 (1.71%) |
|  |  | Cardiovascular | n<5 (0.02%) | n<5 (0.02%) | n<5 (0.02%) | n<5 (0.02%) |
|  |  | Digestive | 685 (2.18%) | 703 (2.81%) | 466 (1.64%) | 381 (1.82%) |
|  |  | Endocrine | n<5 (0.02%) | 7 (0.03%) | 11 (0.04%) | 9 (0.04%) |
|  |  | Immune & haematological | 229 (0.72%) | 229 (0.91%) | 165 (0.58%) | 112 (0.53%) |
|  |  | Nervous | 32 (0.10%) | 25 (0.10%) | 21 (0.07%) | 9 (0.04%) |
|  |  | Other | 158 (0.50%) | 131 (0.52%) | 113 (0.40%) | 62 (0.29%) |
|  |  | Reproductive | 809 (2.61%) | 616 (2.48%) | 148 (0.52%) | 151 (0.71%) |
|  |  | Respiratory | 496 (1.57%) | 429 (1.70%) | 260 (0.91%) | 190 (0.90%) |
|  |  | Skeletal | n<5 (0.02%) | n<5 (0.02%) | n<5 (0.02%) | n<5 (0.02%) |
|  |  | Unknown primary | 103 (0.32%) | 89 (0.35%) | 92 (0.32%) | 83 (0.39%) |
|  |  | Urinary | 256 (0.81%) | 229 (0.91%) | 107 (0.37%) | 63 (0.30%) |

*Denominators:
3 months: all patients
 3 months – 5 years: only those with no outcome at 3 months following index
> 5 years: only those with no outcomes up to and including 5 years following index

Table S7. Adjusted model estimates of all outcomes.

Adjusted model estimates of all outcomes: cancer death, any cancer incidence, cancer incidence by anatomical region and cancer incidence by organ system.
Models adjusted for age, diabetes status, hypertension status and smoking status. Model of digestive system cancer outcome was additionally adjusted for alcohol drinking status.

|  | | | **<= 3 months** | | | **3 months to 5 years** | | | **> 5 years** | | | | |  |
| --- | --- | --- | --- | --- | --- | --- | --- | --- | --- | --- | --- | --- | --- | --- |
|  |  |  | **^a^Estimate (95% CI)** | **p-value** | | **^b^Estimate (95% CI)** | **p-value** | | **^b^Estimate (95% CI)** | **p-value** | | |  |  |
| **Men** | | | | | | | | | | | |  |  |  |
|  | Cancer death | Cancer death | 9.72 (7.28, 13.0) | | <0.01 | 1.35 (1.27, 1.43) | | <0.01 | 1.11 (1.03, 1.19) | | <0.01 | | | |
|  | Cancer incidence | Cancer incidence | 4.74 (4.18, 5.39) | | <0.01 | 1.02 (0.98, 1.07) | | 0.323 | 1.02 (0.96, 1.08) | | 0.544 | | | |
|  | Body Region | Head and neck | 4.17 (1.98, 8.80) | | <0.01 | 1.12 (0.90, 1.40) | | 0.296 | 1.00 (0.76, 1.32) | | 0.983 | | | |
|  |  | Chest | 8.00 (6.05, 10.6) | | <0.01 | 1.02 (0.93, 1.12) | | 0.708 | 1.00 (0.88, 1.13) | | 0.997 | | | |
|  |  | Abdomen | 4.88 (3.70, 6.44) | | <0.01 | 1.14 (1.04, 1.26) | | <0.01 | 1.15 (1.01, 1.31) | | 0.038 | | | |
|  |  | Pelvis | 2.75 (2.23, 3.39) | | <0.01 | 0.90 (0.84, 0.97) | | <0.01 | 0.93 (0.85, 1.02) | | 0.115 | | | |
|  |  | Other | 5.69 (3.71, 8.73) | | <0.01 | 1.09 (0.95, 1.25) | | 0.222 | 1.12 (0.94, 1.34) | | 0.200 | | | |
|  |  | Unknown primary | 7.57 (3.99, 14.3) | | <0.01 | 1.15 (0.93, 1.42) | | 0.194 | 1.07 (0.80, 1.44) | | 0.643 | | | |
|  | Organ System | Digestive | 4.15 (3.26, 5.29) | | <0.01 | 1.01 (0.93, 1.10) | | 0.756 | 1.16 (1.04, 1.29) | | 0.010 | | | |
|  |  | Immune & haematological | 6.03 (3.87, 9.39) | | <0.01 | 1.09 (0.94, 1.27) | | 0.252 | 1.18 (0.98, 1.43) | | 0.084 | | | |
|  |  | Reproductive | 2.22 (1.69, 2.91) | | <0.01 | 0.92 (0.85, 1.00) | | 0.065 | 0.89 (0.80, 0.99) | | 0.035 | | | |
|  |  | Respiratory | 9.07 (6.70, 12.3) | | <0.01 | 1.03 (0.93, 1.14) | | 0.555 | 0.95 (0.83, 1.09) | | 0.471 | | | |
|  |  | Urinary | 4.38 (2.96, 6.47) | | <0.01 | 0.98 (0.85, 1.13) | | 0.758 | 0.97 (0.80, 1.17) | | 0.761 | | | |
|  |  | Other | 2.73 (1.44, 5.19) | | <0.01 | 1.08 (0.90, 1.31) | | 0.398 | 0.91 (0.72, 1.15) | | 0.423 | | | |
|  |  | Unknown primary | 7.57 (3.99, 14.3) | | <0.01 | 1.15 (0.93, 1.42) | | 0.194 | 1.07 (0.80, 1.44) | | 0.643 | | | |
| **Women** |  |  |  | |  |  | |  |  | |  | | | |
|  | Cancer death | Cancer death | 7.51 (5.70, 9.88) | | <0.01 | 1.25 (1.17, 1.34) | | <0.01 | 1.03 (0.94, 1.13) | | 0.491 | | | |
|  | Cancer incidence | Cancer incidence | 4.38 (3.81, 5.03) | | <0.01 | 1.06 (1.01, 1.12) | | 0.018 | 0.96 (0.89, 1.03) | | 0.287 | | | |
|  | Body Region | Head and neck | 2.98 (1.09, 8.10) | | 0.033 | 1.14 (0.85, 1.52) | | 0.382 | 0.64 (0.42, 0.98) | | 0.042 | | | |
|  |  | Chest | 3.89 (3.14, 4.82) | | <0.01 | 1.03 (0.94, 1.11) | | 0.552 | 0.97 (0.87, 1.09) | | 0.597 | | | |
|  |  | Abdomen | 5.19 (3.78, 7.14) | | <0.01 | 1.07 (0.96, 1.19) | | 0.229 | 1.01 (0.86, 1.18) | | 0.914 | | | |
|  |  | Pelvis | 3.98 (2.91, 5.43) | | <0.01 | 1.05 (0.93, 1.17) | | 0.457 | 0.99 (0.84, 1.17) | | 0.895 | | | |
|  |  | Other | 6.08 (3.82, 9.68) | | <0.01 | 1.11 (0.93, 1.32) | | 0.254 | 0.85 (0.67, 1.09) | | 0.202 | | | |
|  |  | Unknown primary | 6.50 (3.65, 11.6) | | <0.01 | 1.18 (0.97, 1.43) | | 0.104 | 1.15 (0.85, 1.57) | | 0.365 | | | |
|  | Organ System | Breast | 1.83 (1.34, 2.50) | | <0.01 | 1.00 (0.90, 1.12) | | 0.945 | 1.03 (0.89, 1.19) | | 0.716 | | | |
|  |  | Digestive | 5.26 (3.98, 6.95) | | <0.01 | 1.06 (0.96, 1.16) | | 0.246 | 1.00 (0.87, 1.15) | | 0.978 | | | |
|  |  | Immune & haematological | 5.64 (3.53, 9.02) | | <0.01 | 1.09 (0.91, 1.30) | | 0.367 | 0.84 (0.65, 1.08) | | 0.178 | | | |
|  |  | Reproductive | 3.15 (2.01, 4.92) | | <0.01 | 1.03 (0.87, 1.22) | | 0.751 | 1.23 (0.98, 1.54) | | 0.080 | | | |
|  |  | Respiratory | 7.32 (5.18, 10.4) | | <0.01 | 1.06 (0.92, 1.21) | | 0.433 | 0.88 (0.72, 1.06) | | 0.184 | | | |
|  |  | Urinary | 4.87 (2.49, 9.52) | | <0.01 | 1.08 (0.87, 1.35) | | 0.478 | 0.74 (0.54, 1.02) | | 0.070 | | | |
|  |  | Other | 2.22 (1.17, 4.20) | | 0.015 | 0.98 (0.79, 1.23) | | 0.888 | 0.68 (0.49, 0.93) | | 0.018 | | | |
|  |  | Unknown primary | 6.50 (3.65, 11.6) | | <0.01 | 1.18 (0.97, 1.43) | | 0.104 | 1.15 (0.85, 1.57) | | 0.365 | | | |

^a^<= 3 months (short-term follow-up) estimates represent risk ratios from adjusted Poisson regression.
^b^3 months to 5 years (medium-term) and > 5 years (long-term) estimates represent sub-distribution hazard ratios from adjusted Fine-Gray regression.


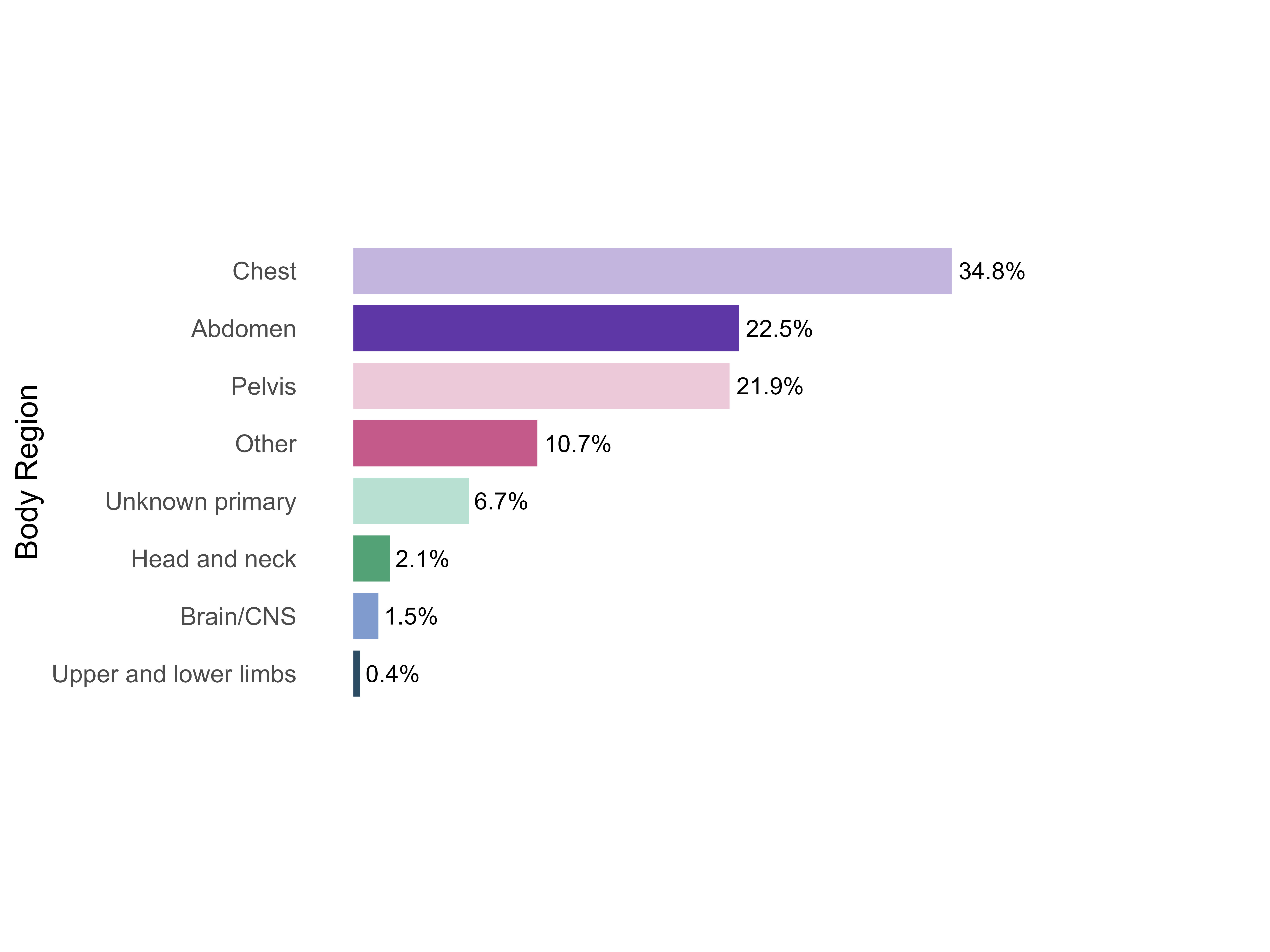


Figure S2. Distribution of cancer sites by body region in patients with new-onset atrial fibrillation that were diagnosed with cancer within 3 months. Percentages represent the proportion of cancer sites by body region in patients with new-onset atrial fibrillation (AF) that were diagnosed with cancer within 3 months of their AF diagnosis. Nearly 80% of diagnosed cancers within 3 months of new-onset AF were located in the chest, abdomen and pelvis.


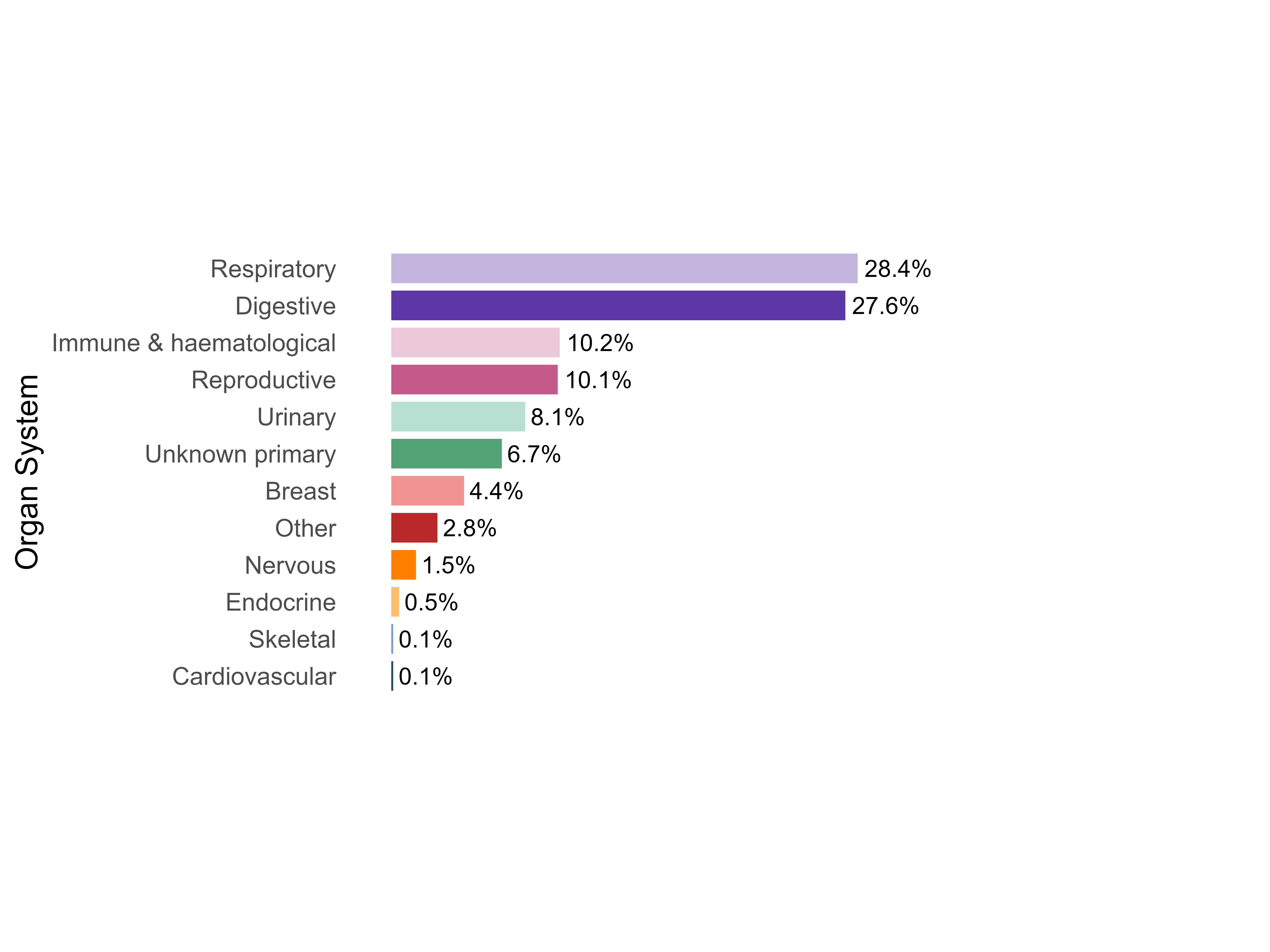


Figure S3. Distribution of cancer sites by organ system in patients with new-onset atrial fibrillation that were diagnosed with cancer within 3 months. Percentages represent the proportion of cancer sites by organ system in patients with new-onset atrial fibrillation (AF) that were diagnosed with cancer within 3 months of their AF diagnosis. Nearly 76% of diagnosed cancers within 3 months of new-onset AF were involved in the respiratory, digestive, immune and haematological, and reproductive systems.

Table S8. Mendelian Randomisation analyses estimates.

| **Outcome** | **Sample size** | **n Cases** | **Model** | **OR (95%CI)** | **p-value** | **n variants** |
| --- | --- | --- | --- | --- | --- | --- |
| Lung Cancer | 85,716 | 29,266 | ^a^IVW | 1.00 (0.96-1.03) | 0.849 | 302 |
| Breast Cancer | 247,173 | 133,384 | ^b^MR-Egger | 0.95 (0.81-1.11) | 0.530 | 312 |
| Colorectal Cancer | 185,998 | 73,149 | ^a^IVW | 1.02 (0.99-1.04) | 0.126 | 299 |

^a^IVW = inverse-variance weighted
^b^MR-Egger = Mendlian Randomisation – Egger
